# Phosphodiesterase-5 inhibition collaborates with vaccine-based immunotherapy to reprogram myeloid cells in pancreatic ductal adenocarcinoma

**DOI:** 10.1172/jci.insight.179292

**Published:** 2024-08-06

**Authors:** Nicole E. Gross, Zhehao Zhang, Jacob T. Mitchell, Soren Charmsaz, Alexei G. Hernandez, Erin M. Coyne, Sarah M. Shin, Diana Carolina Vargas Carvajal, Dimitrios N. Sidiropoulos, Yeonju Cho, Guanglan Mo, Xuan Yuan, Courtney Cannon, Jayalaxmi Suresh Babu, Melissa R. Lyman, Todd Armstrong, Luciane T. Kagohara, Katherine M. Bever, Dung T. Le, Elizabeth M. Jaffee, Elana J. Fertig, Won Jin Ho

**Affiliations:** 1 Department of Oncology, Sidney Kimmel Comprehensive Cancer Center;; 2 Convergence Institute;; 3 Department of Genetic Medicine;; 4 Bloomberg-Kimmel Institute for Cancer Immunotherapy; and; 5 Department of Biomedical Engineering, Johns Hopkins University School of Medicine, Baltimore, Maryland, USA.; 6 Department of Applied Mathematics and Statistics, Johns Hopkins University Whiting School of Engineering, Baltimore, Maryland, USA.

**Keywords:** Immunology, Oncology, Cancer immunotherapy, Immunotherapy, Phosphodiesterases

## Abstract

Pancreatic ductal adenocarcinoma (PDAC) is highly lethal and resistant to immunotherapy. Although immune recognition can be enhanced with immunomodulatory agents including checkpoint inhibitors and vaccines, few patients experience clinical efficacy because the tumor immune microenvironment (TiME) is dominated by immunosuppressive myeloid cells that impose T cell inhibition. Inhibition of phosphodiesterase-5 (PDE5) was reported to downregulate metabolic regulators arginase and inducible NOS in immunosuppressive myeloid cells and enhance immunity against immune-sensitive tumors, including head and neck cancers. We show for the first time to our knowledge that combining a PDE5 inhibitor, tadalafil, with a mesothelin-specific vaccine, anti–programmed cell death protein 1, and anti–cytotoxic T lymphocyte–associated protein 4 yields antitumor efficacy even against immune-resistant PDAC. To determine immunologic advantages conferred by tadalafil, we profiled the TiME using mass cytometry and single-cell RNA-sequencing analysis with Domino to infer intercellular signaling. Our analyses demonstrated that tadalafil reprograms myeloid cells to be less immunosuppressive. Moreover, tadalafil synergized with the vaccine, enhancing T cell activation including mesothelin-specific T cells. Tadalafil treatment was also associated with myeloid/T cell signaling axes important for antitumor responses (e.g., *Cxcr3*, *Il12*). Our study shows that PDE5 inhibition combined with vaccine-based immunotherapy promotes pro-inflammatory states of myeloid cells, activation of T cells, and enhanced myeloid/T cell crosstalk to yield antitumor efficacy against immune-resistant PDAC.

## Abstract 2

Phosphodiesterase-5 inhibition collaborates with a mesothelin-specific vaccine and immune checkpoint inhibitors to yield antitumor efficacy through myeloid and lymphocyte modulation in pancreatic ductal adenocarcinoma.

## Graphical abstract

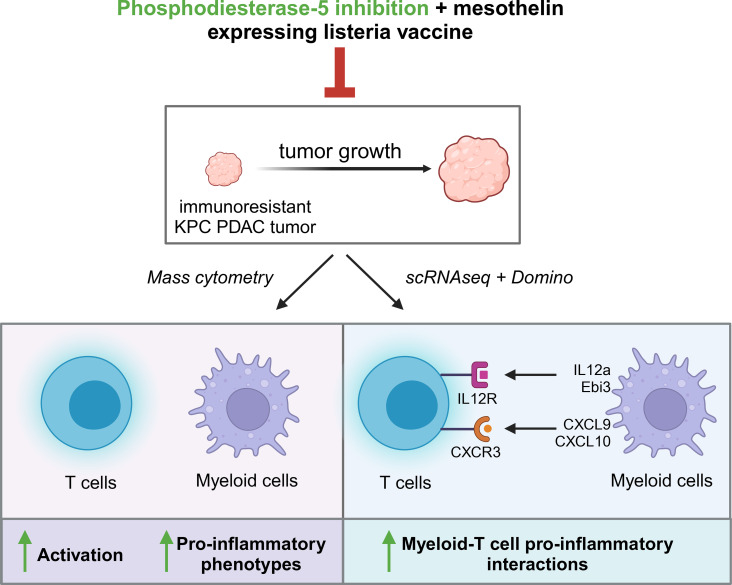

## Introduction

Pancreatic ductal adenocarcinoma (PDAC) is the third leading cause of cancer deaths in the United States and is expected to become the second leading cause by 2030 (1, 2). Most patients with PDAC present with advanced disease, become rapidly resistant to standard-of-care cytotoxic chemotherapies, and rarely respond to radiotherapies and immunotherapies. PDAC’s marked resistance to immunotherapies is attributed to its “cold” tumor immune microenvironment (TiME), which consists of an abundance of immunosuppressive myeloid subtypes, e.g., tumor-associated macrophages (TAMs), granulocytes, other immunosuppressive myeloid cells, T regulatory cells, and cancer-associated fibroblasts (CAFs). Several studies demonstrated that myeloid cell density correlates with poor clinical outcomes in PDAC, including patients treated with a GM-CSF–secreting pancreatic cancer cell vaccine (3–5). Indeed, myeloid cell subtypes exert multiple pro-tumor effects and are known to support PDAC progression, angiogenesis, and metastasis and to suppress antitumor immune responses following treatment (6–10).

The recognized immunosuppressive role of cancer-associated myeloid cells led to efforts to target these cells in conjunction with immunotherapies. In addition to simply trying to eliminate them or block their recruitment (11–14), several approaches aim to reprogram their functions to be less pro-tumor and more pro-inflammatory. One potential approach is to inhibit phosphodiesterase type 5 (PDE5) (15), the hydrolytic enzyme of cyclic guanosine monophosphate (cGMP), which is an important regulator of cGMP-mediated processes, including immune cell metabolism. PDE5 modulates the myeloid expression of arginase-1 (ARG1) and inducible nitric oxide synthase (iNOS) (16–19), and Serafini et al. demonstrated that PDE5 inhibition downregulates IL-4 receptor-α (IL-4Ra), iNOS, and ARG1 expression leading to increased T cell effector activity in preclinical models (17). These findings have been recapitulated in multiple clinical studies in head and neck squamous cell carcinoma and multiple myeloma (18–20). Inhibiting PDE5 is of specific translational interest due to the availability of FDA-approved PDE5 inhibitors (e.g., sildenafil, tadalafil, vardenafil, and avanafil) and their well-established safety profiles. Despite the ongoing progress in studying PDE5 inhibition for treating cancers, the effects of PDE5 inhibition have yet to be evaluated in immune-resistant cancers such as PDACs. The characterization of induced immune responses has also largely been limited. Furthermore, the effects of PDE5 inhibition on immune checkpoint inhibitors (ICIs) and cancer-specific vaccination responses remain unclear.

Due to the high prevalence and demonstrated role of myeloid cells in PDAC resistance to therapy, we hypothesized that PDE5 inhibition will reprogram the immunosuppressive myeloid compartment within the PDAC TiME to collaborate with vaccine and ICI therapy and improve antitumor efficacy. In this study, we investigated the efficacy and resulting immunologic changes following PDE5 inhibition given in combination with a live-attenuated mesothelin expressing listeria vaccine (msln-listeria), which we are developing clinically (21–24), and ICIs in preclinical models of PDAC. For more clinical relevance, we used syngeneic tumors driven by mutant *Kras* (*mKras*) and *Trp53* (KPC) that are known to resist a multiagent combination of chemotherapy plus immunotherapy. To enable time-sensitive and robust evaluation, we tracked and harvested subcutaneous KPC tumors treated with different immunotherapeutic combinations given with PDE5 inhibition. To undertake a detailed exploration of the effects of PDE5 inhibition on the TiME, we employed high-parameter, single-cell approaches, i.e., mass cytometry and single-cell RNA-Seq (scRNA-Seq), to deeply profile the immune cells and their molecular phenotypes. Our study reveals that PDE5 inhibition synergizes with immunotherapies to improve antitumor efficacy in a highly resistant PDAC mouse model, reprograms the myeloid compartment toward a pro-inflammatory state — i.e., away from immunosuppressive phenotypes — increases T cell activation, and achieves significant efficacy in a short time.

## Results

### PDE5 is a relevant target in human PDAC, and expression correlates with inflammatory gene signatures.

To first characterize PDE5 in human PDAC, we assessed *PDE5* expression in The Cancer Genome Atlas (TCGA) by comparing across 19 cancer types along with 18 normal tissue types. While normal pancreas tissues were among the top 50% of normal tissue types examined for *PDE5* expression, PDAC tissue samples (PAAD) were among the top 26.3% of cancer types examined (Supplemental Figure 1A; supplemental material available online with this article; https://doi.org/10.1172/jci.insight.179292DS1). To explore whether *PDE5* expression is involved in inflammation in PDAC, we calculated Pearson’s correlation coefficients between *PDE5* and genes related to immune signatures, fibroblasts, and endothelial cells (Supplemental Figure 1B). *PDE5* expression was significantly correlated with *CD8A*, *CXCR2*, *DCN*, *NOTCH2*, and *PTPRC*, as well as with *ACTA2*, *MRC1*, *NOTCH1*, and *PECAM1*. We also validated PDE5 protein expression using IHC in matched primary and liver metastatic PDAC samples from research autopsy. We observed that PDE5 expression was heterogenous across patients and that the expression was generally higher in pancreas samples compared with matched liver samples (Supplemental Figure 1C). Together, these data suggest that PDE5 is relatively high in expression in PDACs compared with other cancers, associated with inflammatory signatures, and heterogeneous in expression among patients and therefore may be a relevant immunotherapeutic target in at least a subset of PDAC.

### PDE5 inhibition synergizes with immunotherapies to shift myeloid and lymphoid compartments toward pro-inflammatory states and reduce tumor growth in PDAC mouse models.

Upon characterizing the potential relevance of PDE5 as an immunotherapeutic target for PDAC, we next directly studied the immunologic effects of combining PDE5 inhibition with immunotherapies in preclinical models. First, to determine how PDE5 inhibition affects immune-mediated responses in PDAC with or without anti–programmed cell death protein 1 (anti–PD-1) therapy, a mainstay ICI, we used a mouse model that has been previously described to be highly enriched for immune effector cells (*Kras^G12D^* and *Trp53^R172H^* mutant murine PDAC cell line, KPCY 2838c3) (25). C57BL/6J syngeneic mice were injected subcutaneously with KPCY 2838c3 cells 3 days before treatment onset. Mice were treated 3 times per week intraperitoneally with either tadalafil or vehicle and 2 times per week intraperitoneally with either anti–PD-1 or isotype (Supplemental Figure 2A). As expected, tumors did not respond to single-agent therapies, but the combination of tadalafil and anti–PD-1 yielded modest antitumor efficacy (Supplemental Figure 2B). Next, tumors were harvested at 21 days from inoculation, dissociated, and stained for analysis by cytometry by time-of-flight (CyTOF). Samples were stained with a panel of 37 antibodies in addition to 10 channels dedicated to barcodes allowing for the interrogation of lymphoid and myeloid subsets in conjunction with functional markers (Supplemental Table 1). After acquisition, the data were debarcoded and subsequently underwent unsupervised clustering using the Flow Self-Organizing Map (FlowSOM) algorithm, yielding 40 metaclusters that were further annotated into 24 final clusters (Supplemental Figure 2C).

When compared with anti–PD-1 alone, the addition of tadalafil showed a significant reduction in Mac_III, the most abundant F4/80^+^ macrophage cluster and the only MHC class II–negative (MHCII^–^) macrophage cluster (Supplemental Figure 2D). Although tadalafil did not reduce macrophage subset abundances, functional marker analysis within each annotated cluster measured by mean metal intensities (MMIs) uncovered increased CD137 expression in most macrophage clusters, which could contribute to antitumor activity (26), and trends toward decreased CD206 expression in all MHCII^+^ macrophage clusters (Supplemental Figure 2E). In DC clusters, tadalafil increased CD40, Ki67, and CD137 expression, suggesting improved activation and proliferation of DCs (Supplemental Figure 2F). Last, tadalafil also enhanced activation of T cell subsets, increasing the expression of CD25, CD44, OX40, eomesodermin (EOMES), cytotoxic T lymphocyte–associated protein 4 (CTLA-4), or programmed cell death ligand 1 (PD-L1) in specific CD4^+^ T helper (Th) and CD8^+^ cytotoxic T (Tc) cell clusters (Supplemental Figure 2G). Tadalafil-treated groups also had decreased KLRG1 expression, a marker for cellular senescence, in Th and Tc cells (Supplemental Figure 2G). Changes in CD25, OX40, CTLA-4, and KLRG1 within Th cells were particularly notable in tadalafil plus anti–PD-1 combination only. Overall, tadalafil treatment modulated the PDAC TiME T cell and monocyte populations toward pro-inflammatory states when given with anti–PD-1.

Upon verifying the functional shifts in TAMs, DCs, and T cells in the KPCY 2838c3 mouse model, we next tested the efficacy of tadalafil in more immunosuppressive and clinically relevant PDAC models. Like KPCY 2838c3, KPCY 6419c5 and KPCY 6422c1 are KPC-derived clones but create a more immunosuppressive, myeloid-dominated TiME, which has been shown to resist treatment with gemcitabine, abraxane, CD40 agonist, PD-1 inhibition, and CTLA-4 inhibition (25). Before testing efficacy, we validated the contrasting TiMEs of these models and characterized their tumor growth rates by generating subcutaneous tumors as described above. Despite similar tumor growth rates, at least in the first 3 weeks (Supplemental Figure 3A), KPCY 2838c3 tumors had the highest infiltration of CD3^+^ T cells and similar infiltration of F4/80^+^ TAMs compared with KPCY 6419c5 and KPCY 6422c1 (Supplemental Figure 3B). To further characterize these cell lines, we performed immunoblotting for mesothelin (MSLN), a tumor-associated antigen in PDAC, and PDE5, the target of tadalafil, on KPCY 6422c1, KPCY 6419c5, and KPCY 2838c3 cell lysates. Although KPCY 6419c5 had the highest expression of MSLN, all cell lines were comparably positive for PDE5 (Supplemental Figure 3C).

**Figure 1:**
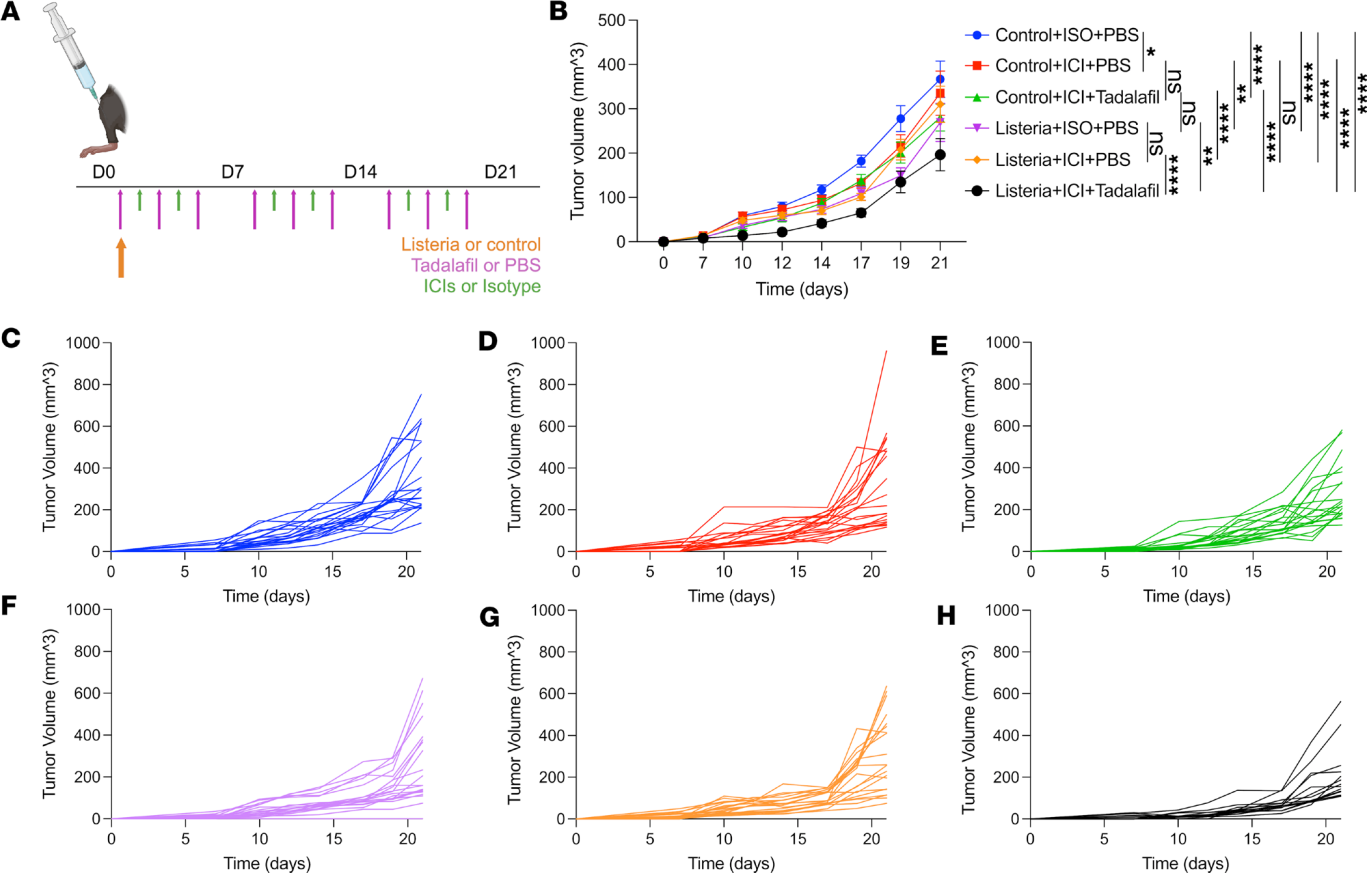
The addition of tadalafil to ICIs and mesothelin expressing listeria vaccine diminishes tumor growth. (**A**) Mice were injected with KPCY 6419c5 cells subcutaneously in the right hind limb on day 0, then vaccinated with mesothelin expressing listeria vaccine or control vaccine on day 3. Mice were treated intravenously with tadalafil or PBS 3 times per week and intraperitoneally with anti–PD-1 and anti–CTLA-4 or respective isotypes 2 times per week until day 26. (**B**) Tumor volumes (mm^3^) were measured 3 times per week starting on day 10 and plotted over time for each treatment group. Plot is representative of 2 independent runs. Mean ± SEM (*n* = 20, except for group 6 with *n* = 15). *P ≤ 0.05, **P ≤ 0.001, ****P ≤ 0.0001 by nonlinear regression. (**C**–**H**) Tumor volumes plotted individually by treatment group.

Since the KPCY 6422c1 and KPCY 6419c5 models present a higher barrier to treatment response, we combined tadalafil with dual ICIs (anti–PD-1 and anti–CTLA-4) as well as our msln-listeria vaccine. We opted to test msln-listeria as the vaccine platform based on our previous development for mouse studies (27), validation of MSLN expression in our models, prior clinical experience demonstrating its immunological efficacy (3, 21, 22), and an ongoing in-parallel clinical investigation (ClinicalTrials.gov NCT05014776). First, C57BL/6J mice were injected subcutaneously with KPCY 6419c5 cells 3 days before treatment onset. Mice were vaccinated intravenously with the msln-listeria or control vaccine (ctrl-listeria) on day 3 and treated 3 times per week intraperitoneally with either tadalafil or PBS and 2 times per week intraperitoneally with either ICIs or their respective isotypes (Figure 1A). Resulting tumors were measured twice per week for 21 days, and tumor volumes were plotted over time. In mice receiving ctrl-listeria, ICI-treated mice had significantly reduced tumor growth compared with isotype-treated mice. Similarly, in mice treated with ICI isotypes, msln-listeria reduced tumor growth when compared with ctrl-listeria–treated mice (Figure 1B). While the addition of tadalafil in the context of ICIs with ctrl-listeria did not contribute additional efficacy, tadalafil significantly increased the efficacy of ICIs with msln-listeria, the total combination of which resulted in an average of 50% tumor growth reduction (Figure 1B). This antitumor efficacy is also evident on a per-mouse scale, signifying that the effect is not driven by outliers (Figure 1, C–H).

**Figure 2:**
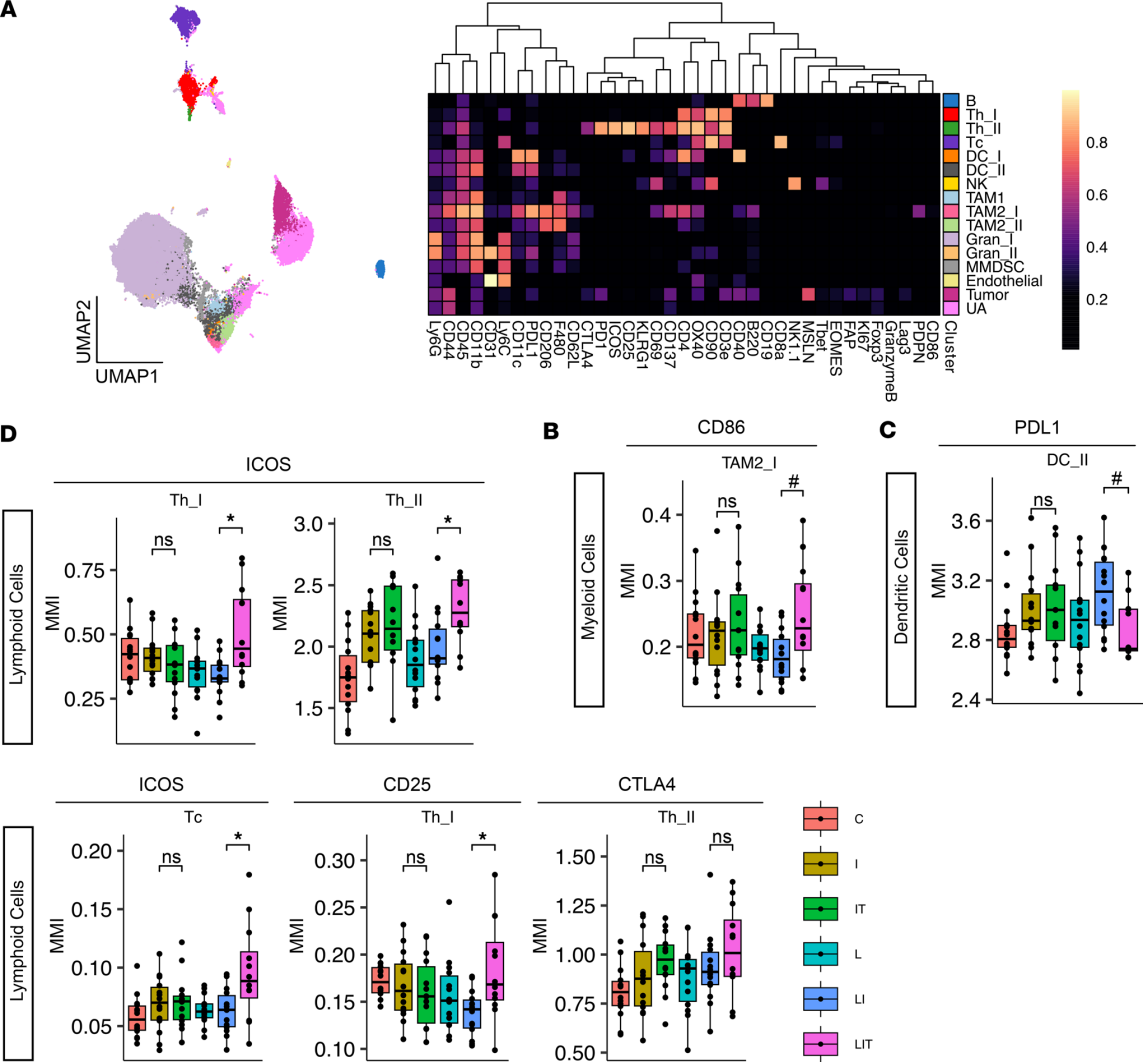
Deep profiling of myeloid, dendritic, and lymphoid cells by CyTOF in subcutaneous KPCY 6419c5 model. (**A**) Uniform manifold approximation and projection (UMAP) and heatmap of final annotated clusters based on relative staining intensities of canonical subtyping markers. UMAP shows 2,000 cells per sample. B, B cell; Th, helper T cell; Tc, cytotoxic T cell; DC, dendritic cell; NK, natural killer cell; TAM, tumor-associated macrophage; Gran, granulocyte; MMDSC, monocytic myeloid-derived suppressor cell; UA, unassigned. (**B**–**D**) Boxplots showing mean metal intensity (MMI) of selected functional markers within selected clusters (*n* = 14). C, control; I, ICIs (anti–PD-1 & anti–CTLA-4); IT, ICIs + tadalafil; L, listeria vaccine; LI, listeria vaccine + ICIs; LIT, listeria vaccine + ICIs + tadalafil. For FDR-adjusted *P* values: *P ≤ 0.05, for unadjusted *P* values: ^#^*P* ≤ 0.05, by 2-tailed Student’s *t* test between groups of interest.

Given the significantly improved immunotherapeutic efficacy with tadalafil plus vaccine and ICIs against this more resistant KPC tumor, we again profiled the tumors using CyTOF using the same staining workflow and analysis pipeline described above. Clustering of the resulting dataset yielded 16 annotated clusters (Figure 2A). Although there were no differences in cell cluster abundances between treatment groups, when interrogating functional marker expression levels, we again found tadalafil-driven changes in myeloid, DC, and lymphoid compartments. Tadalafil plus msln-listeria and ICIs increased the expression of CD86 in F4/80^+^PD-L1^+^CD206^+^ M2-like TAMs, suggesting a shift to a more M1-like phenotype (Figure 2B). Tadalafil also decreased PD-L1 expression in a subset of DCs (F4/80^–^CD11b^+^CD11c^+^PD-L1^hi^CD40^–^) with this combination (Figure 2C). In the lymphoid compartment, tadalafil-treated mice had evidence of improved T cell activation, supported by increased expression of ICOS and CD25, and similar trends in CTLA-4 expression (Figure 2D). Most of the increase in these activation markers occurred in CD3^+^CD4^+^ Th subsets. This Th activation was consistent with the findings in the KPCY 2838c3 model.

When this treatment regimen was repeated in the other immunosuppressive model, KPCY 6422c1, we observed similar efficacy and remodeling in the myeloid and lymphoid compartments (Supplemental Figure 4). Together, these findings suggest that, even in immunosuppressive models, the addition of tadalafil to vaccine and ICI can augment antitumor efficacy by modulating the myeloid compartment to better promote T cell activation.

Next, we evaluated whether the timing and duration of tadalafil treatment with respect to vaccination were critical for efficacy to determine if tadalafil is more important for enhancing the vaccine or ICI component of therapy. To accomplish this, we used the more resistant 6419c5 tumor model and again started treatment 3 days after injection but with an altered treatment schedule. On day 3, all mice were vaccinated with msln-listeria and treated with ICIs twice per week. Group 1 received vehicle for all 3 weeks; group 2 received tadalafil for all 3 weeks; group 3 received tadalafil for the first week and vehicle for the remaining 2 weeks; and group 4 received vehicle for the first week and tadalafil for the remaining 2 weeks. Tadalafil or vehicle treatments were administered 3 times per week (Supplemental Figure 5A). Consistent with the previous studies, the addition of tadalafil to vaccine and ICIs significantly reduced tumor growth in mice. When assessing how early or late administration of tadalafil affects tumor growth, we observed that there is no difference in efficacy when mice are treated early (group 3) or late (group 4) with tadalafil following tumor establishment. When assessing how the duration of tadalafil affects tumor growth, we observed that groups receiving tadalafil for 1 or 2 weeks (groups 3 or 4) had similar responses to the group that received tadalafil for all 3 weeks (group 2) (Supplemental Figure 5, B–F). Taken together, our data suggest that even a shorter period of tadalafil, whether closer to or farther away from the point of vaccination, is sufficient for improved immunotherapeutic efficacy.

### PDE5 abrogation given with vaccine alone maintains antigen-specific CD8^+^ T cell abundance and enhances CD8^+^ T cell activation.

**Figure 3:**
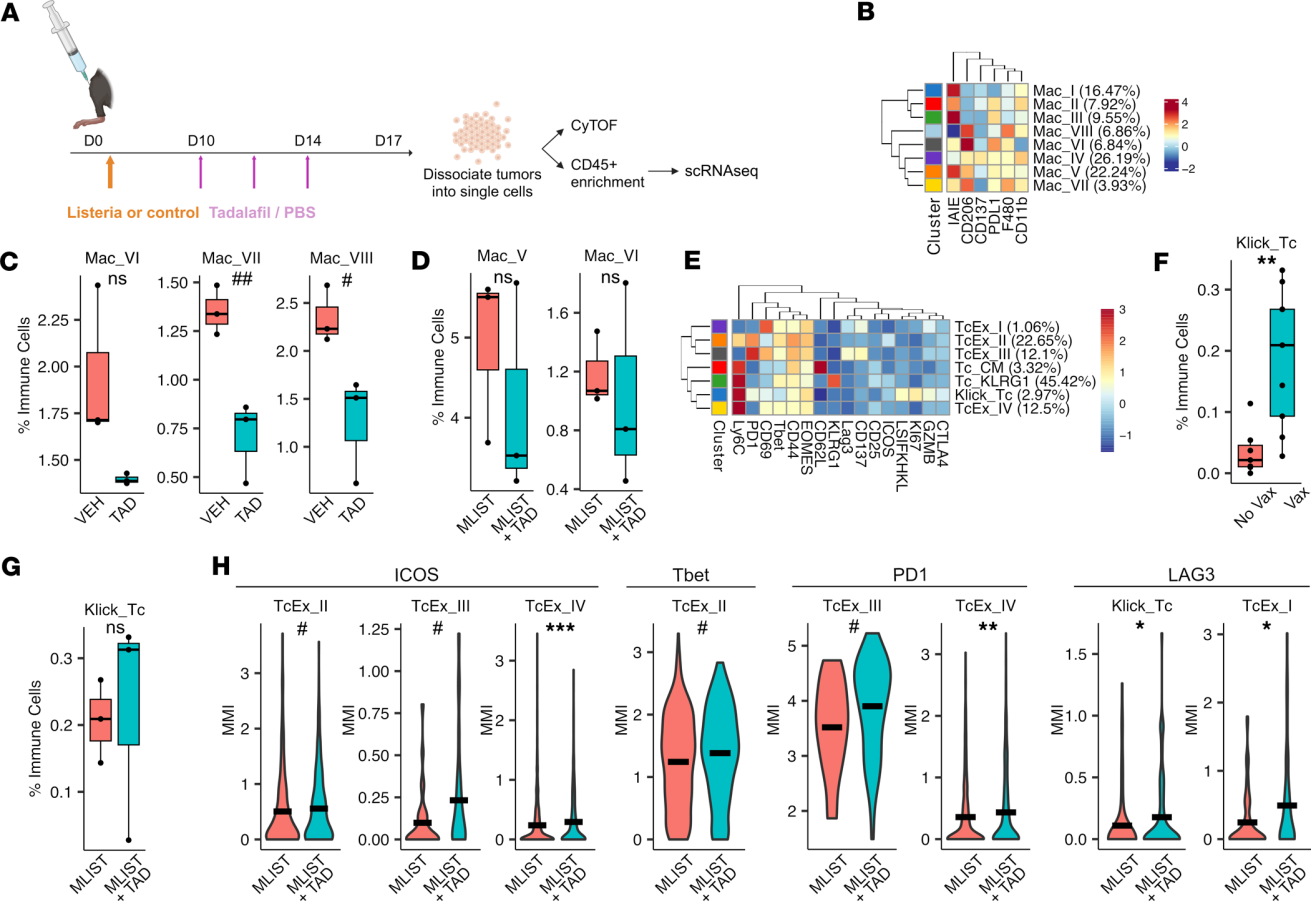
Myeloid and cytotoxic T cell–focused CyTOF profiling of KPCY 6419c5 tumors from mice treated with vaccine and a 7-day delay in tadalafil treatment. (**A**) Mice were injected with KPCY 6419c5 cells subcutaneously in the right hind limb on day 0, then vaccinated with mesothelin expressing listeria vaccine or control vaccine on day 3. Starting on day 10, mice were treated intravenously with tadalafil or PBS 3 times until tumor harvest on day 17. Harvested tumors were dissociated into single cells, which underwent CyTOF staining or CD45^+^ enrichment for scRNA-Seq. (**B**) Clustering heatmap showing macrophage subsets based on relative staining intensities of canonical subtyping markers. Mac, macrophage. (**C** and **D**) Boxplots showing abundances of selected clusters calculated as a percentage of all CD45^+^ cells (*n* = 3). VEH, vehicle; TAD, tadalafil; MLIST, mesothelin listeria; MLIST_TAD, mesothelin listeria + tadalafil. (**E**) Clustering heatmap showing CD8^+^ T cell subsets based on relative staining intensities of canonical subtyping markers. Klick_Tc, Klickmer^+^ cytotoxic T cell; Tc_Ex, exhausted cytotoxic T cell; Tc_CM, central memory cytotoxic T cell; Tc_KLRG1, KLRG1^+^ cytotoxic T cell. (**F** and **G**) Boxplots showing abundances of Klickmer^+^ CD8^+^ T cells calculated as a percentage of all CD45^+^ cells (*n* = 7, 8, for vaccine no and yes groups, respectively) (*n* = 3 for MLIST & MLIST_TAD). (**H**) Violin plots of MMIs of ICOS, T-bet, PD-1, and LAG3 in selected CD8^+^ T cell clusters. For FDR-adjusted *P* values: *P ≤ 0.05, **P ≤ 0.001, ***P ≤ 0.001, for unadjusted *P* values: ^#^*P* ≤ 0.05, ^##^*P* ≤ 0.001 by 2-tailed Student’s *t* test for **C**, **D**, **F**, and **G** and Wilcoxon’s test for **H**.

Given that PDE5 inhibition improved the efficacy of the combined use of vaccine and ICI in association with enhanced immune correlatives, we next evaluated tadalafil-elicited immunological changes shortly after treatment onset. We used the same KPCY 6419c5 resistant tumor model treated with tadalafil in addition to msln-listeria. Since tumors within the first week are too small for sufficient cellular yield, we allowed the tumors to be established longer prior to tadalafil treatment initiation (Figure 3A). Furthermore, to understand the immunologic effects directly mediated by tadalafil with or without msln-vaccine, we did not administer ICI therapy. Specifically, mice were vaccinated with msln- or ctrl-listeria 3 days after tumor inoculation. Mice were then given tadalafil or PBS starting on day 10 and twice more over the subsequent 7 days, after which tumors were harvested and dissociated for analysis (Figure 3A). Dissociated cells were used for CyTOF to evaluate tadalafil effects on cell type abundances and functional changes. Additionally, to study antigen-specific T cell responses, we utilized Klickmers (Immudex) bound by MSLN-derived peptide-MHC monomers and polymers loaded with heavy metal isotopes to allow for detection of T cells bearing MSLN-specific TCRs in conjunction with CyTOF. After acquiring and debarcoding the data, we first analyzed the myeloid compartment to see if the myeloid reprogramming observed previously took place early in the treatment response. Tadalafil treatment alone decreased the abundance of F4/80^+^CD206^+^ macrophage clusters, Mac_VI, Mac_VII, and Mac_VIII (Figure 3, B and C), and showed only moderate trends toward decreased F4/80^+^CD206^+^ macrophage clusters, Mac_V and Mac_VI, when given in combination with msln-listeria (Figure 3, B and D). We then interrogated CD8^+^ T cells to understand how early antigen-specific responses are modulated by tadalafil inhibition. CyTOF-based clustering revealed several CD8^+^ T cell clusters, including Klickmer^+^ Tc cells (Figure 3E). Vaccine-treated mice had significantly higher abundance of MSLN-specific CD8^+^ T cells (Figure 3F). Although the addition of tadalafil did not increase the abundance of antigen-specific T cells, it did not diminish their abundances, verifying that tadalafil does not hamper the early response to vaccine (Figure 3G). Finally, we probed the functional states of CD8^+^ T cells to see how tadalafil might otherwise contribute to early vaccine-induced CD8^+^ T cell responses. We found that the addition of tadalafil increased CD8^+^ T cell activation evidenced by increased ICOS, T-bet, PD-1, and lymphocyte activation gene 3 (LAG3) expression in specific CD8^+^ T cell subsets (Figure 3H). Additionally, antigen-specific T cells also showed increased LAG3 with the addition of tadalafil (Figure 3H). These data provide strong evidence that tadalafil’s immunological effects occur shortly after treatment onset, verifying favorable changes within the TiME that can be further leveraged by the addition of ICIs.

### PDE5 inhibition enhances pro-inflammatory pathways in myeloid cells, augments pro-immune signaling axes, and dampens immunosuppressive signals between myeloid and T cells.

**Figure 4:**
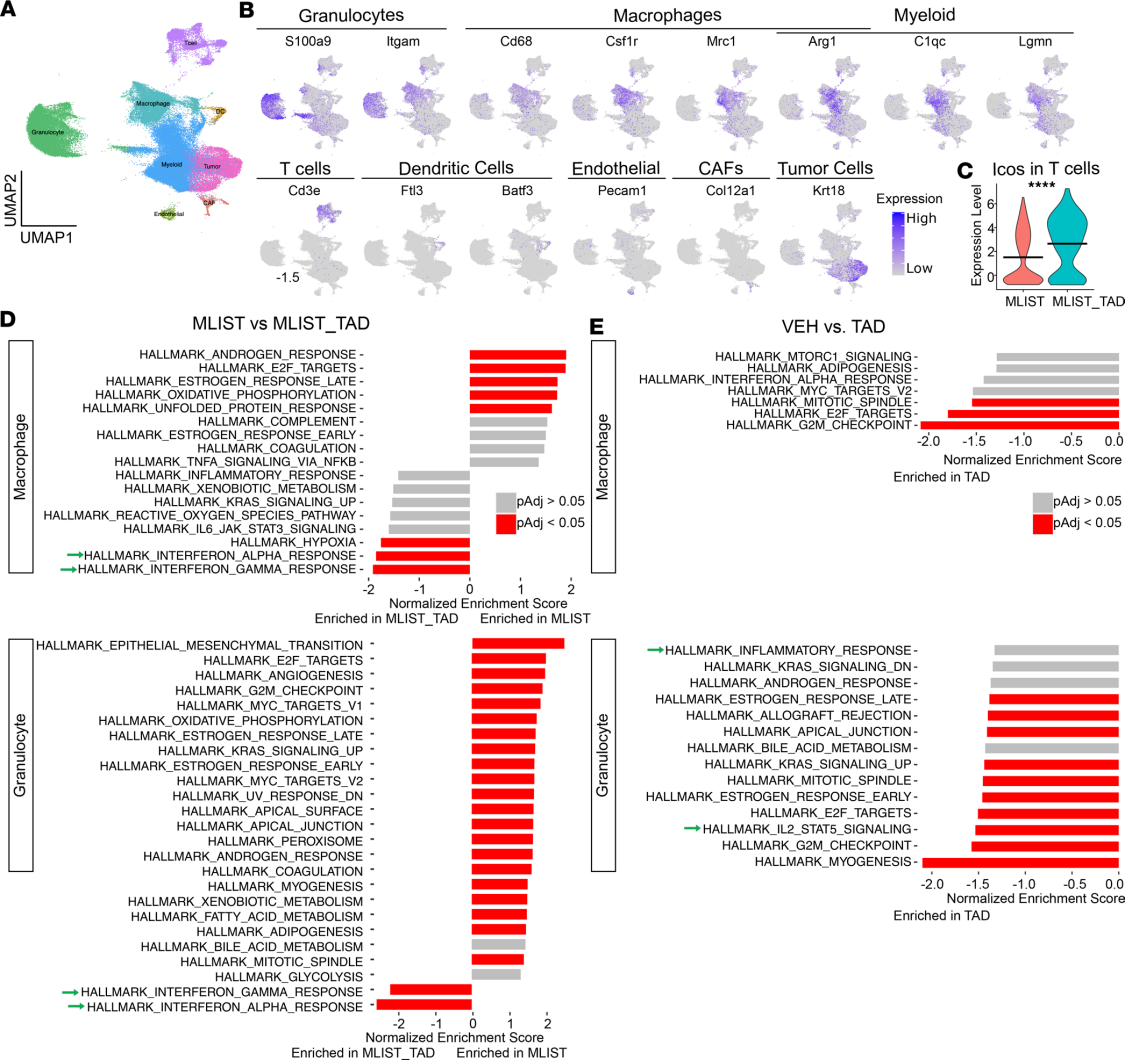
Pro-inflammatory pathways are enriched in macrophage and granulocyte clusters in tadalafil-treated groups. (**A**) UMAP showing 80,543 cells. (**B**) Expression heatmaps showing genes used to annotate granulocyte, macrophage, and myeloid clusters (myeloid clusters); T cells; DCs; endothelial cells; CAFs; and tumor cells. (**C**) Violin plot showing differential expression of *Icos* in T cells at a per-cell level. (**D** and **E**) GSEA showing differentially enriched gene sets from the Hallmark database (*n* = 3). Green arrows are pointing to pathways of interest mentioned in the Results section. VEH, vehicle; TAD, tadalafil; MLIST, mesothelin listeria; MLIST_TAD, mesothelin listeria + tadalafil. ****P ≤ 0.0001 by Wilcoxon’s rank-sum test for **C**.

To understand the early molecular changes in myeloid and lymphoid cells that orchestrate immune responses from tadalafil treatment, we performed scRNA-Seq on 17,000 cells per sample from the same cohort of tumors harvested at 7 days from the onset of treatment with tadalafil with or without msln-listeria (Figure 3A). Preprocessed and clustered data (Supplemental Figure 6) were annotated based on canonical markers (Figure 4, A and B). Differential expression analysis using MAST (28) was performed and validated our CyTOF findings that tadalafil increases T cell activation when given with vaccine. Since CyTOF revealed increases in ICOS expression in T cells, we first sought to validate ICOS upregulation. Indeed, we found significant increases in *Icos* in T cells in the msln-listeria– and msln-tadalafil–treated groups (Figure 4C). To analyze the effects of therapy on the functional states of the cell types in the TiME, we performed gene set enrichment analysis (GSEA) on differential expression statistics between treatment groups to determine tadalafil-mediated signaling pathways using the Molecular Signatures Database (MSigDB) Hallmark gene sets (29). While there were no significantly enriched pathways to indicate T cell activation with tadalafil treatment (Supplemental Figure 7, A and B), there were enhanced pro-inflammatory pathways in myeloid cells. In mice treated with vaccine and tadalafil, both macrophages and granulocytes were significantly enriched in interferon-α and -γ response pathways (Figure 4D). Similarly, without vaccine, macrophages from tadalafil-treated tumors, although not significant, trended toward an enriched interferon-α response, while granulocytes from that group were significantly enriched in the IL-2 and STAT5 signaling pathway (Figure 4E).

**Figure 5:**
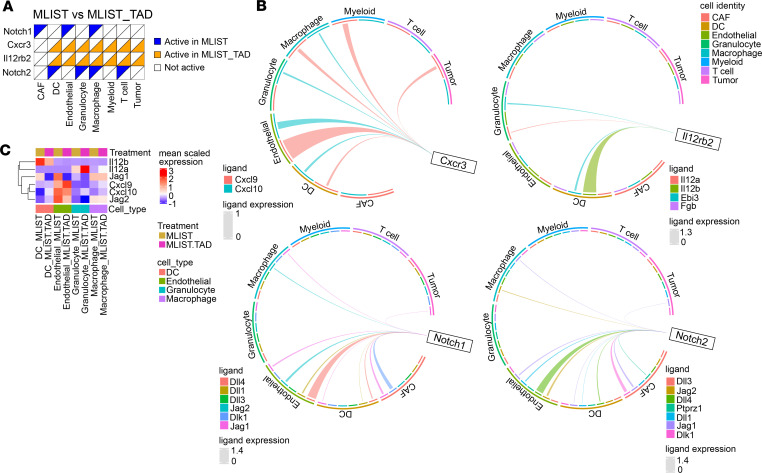
Inflammatory signaling axes are active in vaccine- and tadalafil-treated groups. (**A**) Binary heatmap showing active receptors in cells by treatment group (*n* = 3). (**B**) Circos plots showing ligands of *Cxcr3*, *Il12rb2*, *Notch1*, and *Notch2* and their cellular sources. Thickness of lines correlates to ligand expression levels; thicker lines correspond to higher expression. (**C**) Heatmap showing expression levels of selected ligands in DCs, endothelial cells, granulocytes, and macrophages.

Given the known immunosuppressive role of myeloid cells in regulating T cell responses (4, 6, 30), we examined how tadalafil might influence the immunomodulatory effects of myeloid cells on T cells. To test our hypothesis that tadalafil augments pro-inflammatory myeloid/T cell interactions, we employed Domino to construct inferred intercellular signaling activities based on linked expression of receptors, downstream signaling, transcriptional factors, and regulon genes from the scRNA-Seq dataset (31). In mice treated with the msln-vaccine, CXCR3 signaling, a well-established pathway for recruiting T cells into the TiME, was active in most cell types with tadalafil but not without tadalafil treatment (Figure 5A). Furthermore, myeloid cells (granulocyte, macrophage, myeloid clusters) and endothelial cells were the greatest contributors of the CXCR3 ligands, *Cxcl9* and *Cxcl10* (Figure 5, B and C). IL-12 signaling, namely *IL12rb2*, was also active in most cell types with tadalafil treatment (Figure 5A). DCs and granulocytes were the main contributors of the cognate heterodimer ligand IL-12, which consists of *IL12a* and *IL12b* (Figure 5, B and C), respectively. These findings suggest potential intercellular mechanisms by which tadalafil-mediated myeloid reprogramming results in enhanced T cell activation (e.g., increased CD25, PD-1, ICOS) also indicated by CyTOF data. In addition to enhanced immunostimulatory pathways, tadalafil-treated groups had fewer active pro-tumor signaling pathways. For example, both NOTCH1 and NOTCH2 signaling, which are pathways recently established to play immunosuppressive roles in PDAC, were inactive in macrophages from tadalafil-treated mice but active in macrophages in the absence of tadalafil treatment. Myeloid clusters, endothelial cells, and CAFs were all contributors of NOTCH1/2 ligands, e.g., *Jag1* and *Jag2* (Figure 5, A–C).

While tadalafil treatment without the vaccine did not recapitulate active CXCR3 or IL-12 signaling, it did result in the downregulation of NOTCH1 and NOTCH2 activity (Supplemental Figure 8). In addition, several macrophage-recruiting receptor pathways were inactive with tadalafil treatment. For example, Domino revealed that CSF1R, CCR2, and CCR5 signaling were active across multiple cell types in the control group but not in the tadalafil group (Supplemental Figure 8A). The main ligand contributors for CSF1R were granulocytes (*Csf1*) and CAFs (*Il34*); for CCR2, macrophages (*Ccl7*, *Ccl12*) and CAFs (*Ccl7*); and for CCR5, granulocytes (*Ccl3*, *Ccl4*), macrophages (*Ccl7*, *Ccl12*), and CAFs (*Ccl7*) (Supplemental Figure 8, B and C). In addition to suppression of myeloid-recruiting signaling, tadalafil treatment was associated with downregulated signaling activities that denote M2-like macrophage phenotypes, also consistent with our CyTOF data. Macrophages from the tadalafil-treated group did not exhibit active IL4RA, IL6RA, and MRC1/CD206 signaling, while these receptors were all active in macrophages from the control group (Supplemental Figure 8A) with multiple cell types as major ligand contributors (Supplemental Figure 8, B and C). Tadalafil also downregulated IL10 (*Il10rb*) and adenosine (*Adora2a*) signaling (Supplemental Figure 8, A–C), both of which have been shown to regulate antitumor immune responses (32–35). Finally, tadalafil upregulated lymphotoxin/LTBR signaling in CAFs, endothelial cells, myeloid cells, and tumor cells, with most of the ligand contribution from T cells. Interestingly, lymphotoxin signaling has been associated with immune cell recruitment and tertiary lymphoid structure formation (36–38) (Supplemental Figure 8, A–C). Together, these findings establish that tadalafil in isolation provides substantial reprogramming of the myeloid compartment and when combined with vaccine significantly skews the myeloid/T cell signaling axes toward pro-inflammatory anticancer states.

## Discussion

Myeloid cells are known to be abundant in the PDAC TiME, and their immunosuppressive functions are key contributors to immunotherapeutic resistance (4, 5, 7). Myeloid-targeting therapies for PDAC are urgently needed as components of immune-based approaches to realize the potential of immunotherapy for this cancer, especially those that can be readily implemented in the clinic. Therefore, repurposing FDA-approved drugs, such as PDE5-inhibiting drugs (e.g., tadalafil), is of great interest because of their established safety profiles and availability. While PDE5 inhibition has shown promise in other cancers (15, 17–20), we demonstrated for the first time to our knowledge that PDE5 inhibition collaborates with ICIs and an msln-listeria vaccine to improve efficacy in a highly resistant PDAC mouse model. Rather than depleting or inhibiting myeloid function, PDE5 inhibition reprograms myeloid cells toward a pro–effector T cell response with the potential to reduce tumor growth. These improved efficacy and underlying immunomodulation are sufficiently elicited even with shorter durations of tadalafil treatment. Application of Domino, a cell-to-cell network analysis program for scRNA-Seq data, provided further support for our cytometric evidence of myeloid and T cell immunomodulation, uncovering diminished pro-tumor and enhanced antitumor signaling networks in myeloid cells. These results in integration provide additional insights into the cellular pathways mediated by tadalafil treatment that underlie the observed antitumor efficacy, though causal mechanisms and subsequent functional validations are still necessary.

In addition to the effects of PDE5 inhibition on myeloid suppressive cells as previously reported (17–20), we identified downstream effects on other cell types. We showed that macrophages in particular are reprogrammed with downregulation of pro-tumor M2-like macrophage phenotypes, e.g., CD206^+^, and signaling activities, e.g., CSF1R, CCR2, and CCR5. Furthermore, we found certain gene set pathways, such as interferon signaling, to be significantly enhanced in macrophages only with the addition of vaccine to tadalafil, highlighting the cooperation of PDE5 inhibition and vaccine-mediated immune responses. These changes could be in part due to PDE5 inhibition suppressing IL-4Ra signaling, leading to reduced type 2 cytokine effectors and subsequently M2-like macrophage repolarization. Interestingly, both with and without vaccine, NOTCH1 and NOTCH2 activities were downregulated in macrophages with PDE5 inhibition. While the role of NOTCH signaling in macrophages is not fully understood, there is growing evidence that NOTCH signaling via Jagged1 may drive pro-tumor TAM phenotypes (39). We also observed downregulation of NOTCH signaling in CAFs with PDE5 inhibition plus vaccine. Pro-tumor functions of NOTCH signaling in CAFs have previously been implicated in their regulation of DNA damage response and growth arrest (40). In addition to NOTCH, the role of endothelial cells as major ligand contributors to the immune-recruiting CXCR3 pathway was also notable. These findings are interesting since the role of PDE5 inhibition on endothelial cell function other than vasodilatory effects has not been well characterized. Finally, we saw lymphotoxin (LTBR) signaling increase in many cell types (CAFs, endothelial, myeloid, tumor cells) resulting from PDE5 inhibition. This is yet another inferred pro-immune pathway regulated by PDE5, since lymphotoxin signaling promotes high endothelial venule formation, lymphocyte homing, and tertiary lymphoid structure formation, which correlates with a superior prognosis in some cancers (36–38, 41). Thus, our work establishes that PDE5 inhibition likely facilitates pro-inflammatory changes within myeloid cells and multiple intercellular signaling axes to enable antitumor immunotherapeutic efficacy even against highly immune-resistant PDAC.

We consider the following limitations in this study. Previously, multiple studies focused on the effects of PDE5 inhibition specifically on myeloid suppressive cells and established that PDE5 inhibition downregulates IL4Ra, NOS2, and ARG1 expression in myeloid suppressive cells in several cancer types (17–20). In our model, while we did observe the downregulation of IL-4Ra across myeloid subtypes, including granulocytes, we did not capture significant changes in NOS2 or ARG1. This could be due to technical limitations, e.g., low transcript levels and inability to evaluate the differences in enzymatic activities. While determining the metabolic correlatives of PDE5 inhibition is not the focus of this study, it is still possible then that NOS2- and ARG1-mediated changes in the PDAC TiME would be better captured if metabolites were measured directly. Second, given the necessity to track the tumor growth closely and longitudinally, and the need to flexibly compare the effects of careful timing of the different treatment modalities, we opted to explore the efficacy and TiME changes in the subcutaneous tumor model. Autochthonous tumors result in high variability and unpredictability, limiting sufficient reproducibility in evaluating immunologic changes mediated by precise treatment courses. Orthotopic pancreatic models also restrict our ability to measure tumors closely. Thus, whereas the main purpose of our study was to determine how tumor-infiltrating myeloid subtypes and T cells are reprogrammed by PDE5 inhibition, the reliance on subcutaneous PDAC tumors needs to be considered carefully in context. Additionally, it should be noted that although we validated antitumor efficacy and TiME modulation via CyTOF in multiple models, interrogation of intercellular signaling axes with Domino was performed in a single tumor model and therefore may not fully encompass all the intercellular mechanisms underlying the observed efficacy across models.

Overall, our work warrants studies to further elucidate the exact molecular mechanisms of how the tadalafil-elicited changes within the myeloid cell, endothelial cell, and CAF compartments yield antitumor efficacy, and more importantly, validate the clinical relevance of these changes. While likely not a candidate for single-agent therapy against PDAC, we show that PDE5 inhibition synergizes with immunotherapies to reprogram myeloid cells and can be leveraged to increase efficacy of other treatments. Our work also nominates candidate strategies for improved intervention. For example, our data imply that combinations involving PDE5 inhibition have the potential to be further enhanced by adding antagonists of coinhibitory markers (e.g., LAG3) or agonists of costimulation markers (e.g., ICOS) on T cells. Additionally, targeting other distinct myeloid-modulating pathways such as CD40 agonism could collaboratively reprogram the myeloid compartment to bolster immunotherapeutic efficacy. Since PDE5-inhibiting drugs (e.g., sildenafil, tadalafil, vardenafil, and avanafil) are already FDA approved, are well tolerated, and are widely used in the clinic (e.g., for pulmonary hypertension and erectile dysfunction), they are readily available to be implemented for the treatment of PDAC. The results of our ongoing clinical study (NCT05014776), which similarly investigates the safety and activity of MSLN-targeted listeria vaccine CRS-207, anti–PD-1, anti–CTLA-4, and tadalafil in patients with metastatic PDAC, will provide critical insights to further understand these pathway changes in patients with PDAC. Given the highly heterogeneous expression of PDE5 in human PDAC, our work also encourages IHC quantification of PDE5 in patients enrolled in this study to inform stratification of clinical benefit.

## Methods

### Sex as a biological variable.

The mouse model experiments involving the KPC cell lines utilize both female and male mice given that the different KPC cell lines are derived from both female and male mice and a syngeneic background must be used for immunologic studies. In terms of clinical relevance, there are no known sex-specific phenotypes in PDAC in terms of disease prevalence, prognosis, and treatment response.

### Evaluation of PDE5 expression in human PDACs.

Transcriptome data were obtained from TCGA Data Portal (https://tcga-data.nci.nih.gov/tcga/tcgaHome2.jsp). We utilized transcriptomics data from 19 cancer types and 18 corresponding normal tissue samples. Log_2_-transformed transcripts per million of *PDE5A* were used and visualized across the cancer types with box-and-whisker plots using ggplot2 v3.5.1 package in R v4.3.0. The boxplots were arranged by mean expression value to compare the level of *PDE5A* expression in PAAD against other cancer types. To evaluate the potential of *PDE5A* as a therapeutic target for PAAD, scatterplots were generated to examine the relationship between log_2_-normalized *PDE5A* expression and other inflammatory target RNAs using ggplot2 v3.5.1. Pearson’s method was applied to assess the correlation between *PDE5A* and these RNA targets. PDAC samples from Johns Hopkins were obtained through the Legacy Gift Rapid Autopsy program.

### IHC staining and analysis.

Unstained slides cut from a tissue microarray containing human tissue were baked in a HybEZ oven at 60°C for 1 hour to ensure optimal tissue adhesion. Slides were deparaffinized by immersion in xylene for 20 minutes followed by rehydration in an ethanol gradient (100%, 95%, 80%, and 70%) for 5 minutes each. Subsequently, slides were washed in distilled water for 5 minutes with gentle agitation. Antigen retrieval was performed using a 1:10 dilution of DAKO (10×) solution for human in Maxpar Water. The solution was heated to 105°C in a heat block, and slides were incubated for 60 minutes. After incubation, slides were allowed to cool at room temperature for 10 minutes. To block nonspecific binding, slides were treated with BLOXALL (Vector Laboratories) for 10 minutes at room temperature, followed by washing in PBS-Tween (PBST). Subsequently, slides were incubated with 2.5% normal horse serum for 20 minutes. Excess serum was removed by tapping and was then incubated overnight at 4°C with a primary antibody solution consisting of rabbit monoclonal PDE5A (Abcam EPR24129-95) diluted in 0.5% horse serum. Following primary antibody incubation, slides were washed in PBST and incubated with ImmPRESS polymer reagent (Vector Laboratories) for 30 minutes at room temperature. After 2 additional PBST washes, slides were incubated in ImmPACT DAB substrate (Vector Laboratories) for 2 minutes and 30 seconds to visualize the antigen-antibody complex. Slides were counterstained with hematoxylin for 3–5 minutes, followed by a brief immersion in bluing reagent solution. Finally, slides were air-dried and mounted with DAKO Faramount Aqueous mounting medium, and coverslips were applied. Stained slides were scanned using a Hamamatsu NanoZoomer.

Stained slides were analyzed using HALO software v3.6.4134.396 (Indica Labs). Tumor areas were annotated, and positively stained tissue area was quantified using an optimized algorithm from Indica Labs (Area Quantification v2.4.3). Positive tissue area was divided by tumor area to obtain percentage positive tissue area for respective markers.

### Cell lines and animal models.

For all mouse experiments, male (for KPCY 6422c1) or female (for KPCY 2838c3 and KPCY 6419c5) 7-week-old C57BL/6J mice were purchased from The Jackson Laboratory and housed in the Johns Hopkins University animal facilities. Mice were allowed to acclimate in our mouse facilities for at least 1 week before experimentation.

For KPCY 6422c1, KPCY 2838c3, and KPCY 6419c5 cell lines (Kerafast), cells were thawed and passaged in DMEM-based medium containing 10% FBS, 1% l-glutamine, and 100 U/mL penicillin/streptomycin in 5% CO_2_ at 37°C. On day 0, 2.5 × 10^5^ KPCY 2838c3 or KPCY 6419c5 cells were subcutaneously injected into the right hind limb. Tadalafil was dissolved in DMSO at 20 mg/mL, resuspended with cremophor at 20 mg/mL, and resuspended with 1× PBS. Mice received vehicle or tadalafil (SML1877-50MG, MilliporeSigma; *t_1/2_* ~ 17.5 hours in humans) (42) at 2 mg/kg intraperitoneally 3 times a week. Transient accumulation of enzymatic substrate cGMP and downstream molecular target phosphorylated vasodilator-stimulated phosphoprotein (phospho-VASP) resulting from tadalafil intraperitoneal injection was verified (Supplemental Figure 9). Isotype or CTLA-4 antibody (clone 9H10, BioXCell) and isotype or PD-1 antibody (clone RMP1-14, BioXCell) were administered at 5 mg/kg intraperitoneally twice a week. Mice also received msln-listeria or no-insert listeria intravenously at 5 × 10^5^ colony-forming units. The subcutaneous tumors were harvested on day 21 and weighed to measure tumor volume. Tumor volume was also calculated on day 21 based on caliper-measured major (D) and minor (d) diameters using the following formula: V = 1/2 × D × d^2^. For CyTOF, Klickmer, and RNA-Seq analysis, tumors were processed into single-cell suspensions by physical and enzymatic dissociation (130-096-730, Miltenyi Biotec). Dissociated tumor samples were then filtered through a 70 μm filter (Corning) in RPMI media (Gibco). Cells were resuspended with 40% Percoll in 1× PBS, then underlaid with 80% Percoll in 1× PBS. Cells were spun down and the middle layer containing primarily leukocytes was collected for counting as we have previously described (43). Total cell numbers from individual tumors were counted with a hemocytometer (Hausser Scientific). For CyTOF staining, 2 × 10^6^ cells were plated into each well of a 96-well plate. For Klickmer staining, 1.5 × 10^5^ cells were plated into each well of a 96-well plate. For RNA-Seq staining, at least 1 × 10^6^ cells were enriched for CD45 (130-110-618, Miltenyi Biotec), and 2 × 10^4^ CD45^+^ cells were pipetted into individual tubes.

### Generation of cell lysates.

Subcutaneous tumors were harvested from mice, then snap-frozen using liquid nitrogen immediately following harvest. Frozen tumors were next pulverized into fine powder using a precooled biopulverizer. A total of 20 mg of pulverized tissue was weighed out and aliquoted per sample into 1.5 mL Eppendorf tubes. Pulverized tissue was then suspended in lysis buffer (RIPA + protease/phosphatase inhibitor) and homogenized using a 1.5 mL pestle. The resulting homogenized cell solution was then centrifuged at 17,000*g* at 4°C for 10 minutes. The supernatant was then harvested from each tube and aliquoted to new 1.5 mL tubes. The harvested supernatant represents protein lysates ready for use.

### Immunoblotting

#### cGMP quantification.

A cGMP assay (Revvity catalog 62GM2PEG) was employed to quantify cGMP in cell lysates generated from snap-frozen tissue. Assay was performed based on manufacturer’s protocols. Standards were generated using serial dilution according to the manufacturer’s protocol. Standards, negative controls, and experimental samples were plated in triplicate in a 384–shallow-well plate (Revvity). Lysis/detection buffer was then added to negative control wells while cGMP acceptor reagent working solution was added to all other wells. cGMP donor antibody working solution was then added to all wells. The plate was then sealed and incubated at room temperature for 1 hour. Plate was read on a SpectraMax M5e plate reader (Molecular Devices).

#### Automated immunoblotting.

Immunoblotting for MSLN, PDE5, and phospho-VASP was performed using the Wes Simple Western system (ProteinSimple, Bio-Techne). To perform this capillary-based size separation, manufacturer’s protocols for 12–230 kDa separation module (SM-W004) were used. Briefly, cell lysates from subcutaneous tumors were diluted with 0.1× sample buffer, and then 1 part 5× fluorescent master mix to 4 parts diluted lysate was added to reach a final lysate concentration of 0.33 mg/mL. Lysates were then denatured at 95°C for 5 minutes. Primary antibodies used were 1:250 anti-PDE5 (Cell Signaling Technology catalog 2395S), 1:250 anti-MSLN (Abcam catalog ab187063), and 1:30 anti–phospho-VASP (Cell Signaling Technology catalog 3132). The secondary antibody used for all primaries was anti-rabbit secondary antibody (ProteinSimple, Bio-Techne, Anti-Rabbit Detection Module catalog DM-001). Biotinylated ladder, lysates, antibody diluents, primary antibodies, streptavidin-HRP, secondary antibodies, and luminol-peroxide mix (all ProteinSimple, Bio-Techne) were loaded into the microplate, then centrifuged for 5 minutes at 2,500 rpm. Capillary cartridge and microplate were then loaded into the Wes machine for size-based separation where each capillary contained a unique reaction. ProteinSimple uses photoactivated capture chemistry to immobilize separated proteins on the capillaries, which are then visualized digitally in the Compass Simple Western software (v6.1.0). The software also automatically calculated chemiluminescence intensity (peak heights), area, and signal/noise ratio. To normalize protein expression data, the total protein assay (ProteinSimple, Bio-Techne, catalog DM-TP01) was used according to manufacturer’s protocols. Protein expression was normalized by dividing area under the curve for each protein by total protein area.

#### Antibodies.

The mass cytometry antibodies, isotopes, and concentrations used for experimental staining are listed in Supplemental Table 1. Antibodies with desired channels not available through Standard BioTools were purchased as purified primary antibodies in a BSA- and azide-free formulation from other vendors and conjugated using Maxpar Conjugation Kits according to the manufacturer’s protocol (Standard BioTools). Purified antibodies underwent a buffer exchange protocol with 50 kDa ultrafiltration columns (Amicon) and partial reduction with 4 mM Tris(2-carboxyethyl)phosphine hydrochloride (Thermo Fisher Scientific). Isotopically enriched metals for the desired channels were individually loaded onto the polymers, followed by conjugation of each metal polymer to the appropriate antibody and a wash step. In the wash buffer, antibody concentrations were quantified using NanoDrop (Thermo Fisher Scientific). Stabilization buffer (Candor) with 0.05% (w/v) sodium azide was used to dilute the antibody concentrates. Experimental antibody concentration was identified by testing 3–5 concentrations on positive and negative controls to identify optimal signal with minimal spillover. Additional details for custom antibody conjugation have previously been described in more detail (43).

#### Mass cytometry staining and acquisition.

Immune cells were dissociated nonenzymatically in Maxpar PBS (Standard BioTools) with 2 mM EDTA. Cells were then stained for viability using Cell-ID Cisplatin (Standard BioTools) at 5 mM in Maxpar PBS for 2.5 minutes. After incubation, samples were quenched in RPMI with 10% FBS and 1% of 1× penicillin-streptomycin and then washed twice in Maxpar Cell Staining Buffer (Standard BioTools). Samples were each stained for 25 minutes at room temperature with a unique five-choose-three metal-barcoding scheme for up to 10 barcodes using CD45 and CD98 antibodies at each channel as we have previously described (44). After barcoding, samples were washed with Maxpar Cell Staining Buffer and combined into 10-plexed tubes for subsequent staining. The plexed samples were blocked with anti-mouse Fc block (BD Biosciences) for 10 minutes at room temperature, after which a surface stain antibody cocktail in Maxpar Cell Staining Buffer was added to incubate for an additional 30 minutes. Prior to intracellular staining, a 1:4 dilution of eBio Fixation/Permeabilization Concentrate to eBio Fixation/Permeabilization Diluent (eBioscience) was added to the samples to incubate for 30 minutes at room temperature. Samples were washed twice in 10× eBio Permeabilization buffer (eBioscience) diluted 1:10 using Maxpar Water (Standard BioTools). An intracellular stain antibody cocktail in permeabilization buffer was added to the samples and incubated for 30 minutes at room temperature. After a Maxpar Cell Staining Buffer wash, samples were fixed using 16% Pierce Formaldehyde (Thermo Fisher Scientific) diluted in Maxpar PBS to 1.6% and stored until the day of acquisition for no longer than 1 week. On acquisition day, the DNA Intercalator Cell-ID Intercalator-Rh (Standard BioTools) was diluted 1:500 in Maxpar Fix and Perm Buffer (Standard BioTools) and used to stain the samples for 30 minutes at room temperature. Samples were washed and resuspended in Maxpar Cell Staining Buffer. Additional details for CyTOF staining have previously been described in more detail (43). Prior to acquisition using the Helios mass cytometer (Standard BioTools), samples were washed and resuspended in a 1:10 dilution of EQ Four Element Calibration Beads (Standard BioTools) in Maxpar Cell Acquisition Solution. Mass cytometry data were acquired at the Sidney Kimmel Comprehensive Cancer Center Flow/Mass Cytometry Core of Johns Hopkins University School of Medicine, Baltimore, Maryland, USA.

#### Mass cytometry data preprocessing and debarcoding.

Data from mass cytometry acquisition were generated in FCS format, and CyTOF software (Standard BioTools) v6.7 was utilized for randomization, bead normalization, and bead removal. FlowJo (BD) v10.9 was used to identify single-cell events by gating a population for cell length and 103 rhodium signal. Dead cells were eliminated by gating out events positive for 196 Pt and 198 Pt. Debarcoding of the data was executed by gating events positive for the channel combinations unique to each barcode as we have previously described (44). Each debarcoded sample was then exported as a separate FCS file for analysis in R.

#### Mass cytometry data analysis.

Debarcoded files were analyzed using customized pipelines adapted from Nowicka et al. (45) in R v3.6.2, v4.0.2, or v4.2.3 (all scripts are available on GitHub, see below for URL). Briefly, data underwent unsupervised clustering using the FlowSOM algorithm (46) to identify metaclusters, which are annotated based on canonical markers for immune cell types (e.g., CD45^+^CD3^+^CD4^–^CD8^+^ representing Tc cells). Annotated cell types are further subtyped based on expression of functional markers (e.g., granzyme B and PD-1). Clustering was visualized using a 2-dimensional UMAP dimensionality reduction algorithm (47) showing 2,000 cells per sample. Cluster abundances were calculated as percentages of total CD45^+^ cells. Expression levels for functional markers were quantified as MMIs and were arcsine transformed with a cofactor of 5.

#### Klickmer conjugation and staining.

For mass cytometry experiments utilizing Klickmer staining, an MSLN-specific MHC monomer was used to incorporate the 155Gd-Klickmer marker into the mass cytometry panel. MHC monomers (NIH Tetramer Core Facility) were prediluted and combined with a dilution buffer of 1× Maxpar PBS with 1% FBS for a concentration of 4.78 × 10^–6^ mol/L. Maxpar X8 metal-loaded polymers were prepared according to the manufacturer’s protocol (Standard BioTools) and biotinylated by adding 0.4 mM biotin-PEG thiol (Nanocs) in C buffer (Standard BioTools) in a 2:1 molar ratio, incubating for 90 minutes at 37°C. Dilution buffer was used to wash and achieve a final volume of 100 μL. For the assembled Klickmer, 54.56 μL of dilution buffer, 4 μL of undiluted biotinylated metal-loaded polymer, 5.44 μL of prediluted MHC monomer, and last 32 μL of FITC Klickmer (Immudex) were mixed by pipetting and then incubated in the dark for 30 minutes. The mixture was washed twice with Maxpar PBS in a 60 kDa filter (Amicon) and spun for 10 minutes at 15,000*g*. Maxpar PBS was then added for a final volume of 96 μL. For every 2 × 10^6^ cells, 6 μL of assembled Klickmer was used for staining. The Klickmer stain for mass cytometry was added after the Fc blocking step and was incubated for 10 minutes prior to adding the surface stain antibody cocktail.

#### scRNA-Seq.

Dissociated tumors (for dissociation methods, see above) from mice from 6 treatment groups were pooled to go from *n* = 6 to *n* = 3 per group. Cells were enriched using CD45 microbeads (Miltenyi Biotec), except for cells from g1mus3 and g2mus3 samples because of low cell yields. Cells were then counted and resuspended at a concentration of 1,000 cells per microliter. Library preparations were performed with Chromium Next GEM Single Cell 3′ GEM Kit v3.1 (10x Genomics) with an input of 17,000 cells for a final recovery of 10,000 cells for sequencing. Cells were separated into gel beads in emulsion and single cells were barcoded. cDNA was synthesized and amplified for subsequent sequencing library preparations. Sequencing was performed on the Illumina NovaSeq 6000, following 10x Genomics recommendations, with a coverage of 50,000 reads per cell (Supplemental Table 2).

#### Single cell data pre-processing and clustering.

FASTQ reads were demultiplexed and aligned to the mm10-2020-A reference transcriptome, and unique molecular identifier barcodes were retrieved using Cell Ranger v6.1.2. Single-cell gene expression matrices were analyzed using the Seurat (48) v4.3.0 package in R v4.0.5. Data were pre-processed by filtering cells to include only those with less than 5% mitochondrial RNA and between 200 and 2,500 genes. The raw counts were normalized using SCTransform function (49) in Seurat. Highly variable genes were identified and data were scaled based on mitochondrial gene expression. Using the RunPCA function, cells were projected into their first 50 principal components. Then the RunUMAP function was used to visualize data in 2 dimensions. Unsupervised clustering was performed using the FindClusters function at a resolution of 0.2. The resulting UMAP was visualized and filtered to remove clusters with fewer than 16 cells and any singletons. Differential expression testing was performed using the MAST hurdle model in the FindAllMarkers function with a log fold-change threshold of 0.25 and a minimum fractional expression threshold of 0.25 to identify markers that define clusters (28). Pathway analysis was performed using the R package fsgsea (50) to identify biologically enriched pathways from the MSigDB Hallmark gene sets. Clusters were annotated manually based on canonical markers differentially expressed by each cluster.

Cell-cell communication inference was conducted using Domino (31) v0.1.1. Transcription factor activity quantification was conducted with pySCENIC (51) 0.11.0. Ligand-receptor pairs were obtained from CellPhoneDB (52) v2.0.0. A *P* value threshold of *P* < 0.001 in a 1-sided Wilcoxon’s test of transcription factor activity scores was used to identify active transcription factors in each cell type. A Spearman’s correlation coefficient threshold of *R* > 0.25 was used to determine which receptors and transcription factors were linked based on receptor expression and transcription factor activity scores. Signaling interactions between treatment conditions were compared by running Domino on cells from each treatment group individually and comparing active receptors called by Domino in cell types shared across treatment conditions.

#### Figure generation.

All schematics (Figure 1A, Figure 3A, Supplemental Figure 3A, Supplemental Figure 6A) were created with BioRender.com. Tumor growth curves were created with GraphPad Prism v10.1.0. All other plots were created with various packages as detailed above in R v3.6.2, v4.0.2, v4.0.5, or v4.2.3 or GraphPad Prism v10.1.0. All figures were compiled in Inkscape v1.3.

#### Statistics.

Differential analyses of CyTOF data were performed with 2-tailed, 2-sample Student’s *t* tests between treatment groups of interest using the stat_compare_means function in the ggpubr package v0.6.6. *P* values were FDR adjusted using the p.adjust function in the R Stats package v4.2.3. Nonlinear regression was performed on tumor growth curves in GraphPad Prism v10.1.0. Statistical significance was considered for *P* < 0.05.

#### Study approval.

Individual protocols for the care and maintenance of animals have been approved by Johns Hopkins University School of Medicine Institutional Animal Care and Use Committee. Research autopsy was approved by the Johns Hopkins institutional review board and deemed in accordance with the Health Insurance Portability and Accountability Act. With informed consent, primary and metastatic PDAC samples were harvested as soon as possible after death via an autopsy, codified without any identifiable information, and fixed in formalin for paraffin embedding.

#### Data availability.

Mass cytometry and RNA-Seq analysis scripts are available at the following GitHub repository: https://github.com/wjhlab/Tadalafil-Study (commit ID e05acd8). Data from scRNA-Seq can be found at https://www.ncbi.nlm.nih.gov/bioproject/PRJNA1062825 (accession PRJNA1062825). Data from mass cytometry can be found at https://zenodo.org/records/11478985 Supporting data, including values represented in all figures and *P* values, can be found in the Supporting Data Values file.

## Author contributions

NEG, SC, AGH, EMC, SMS, DCVC, JSB, and TA all contributed to mouse experiments, mass cytometry processing and staining, and data analysis. LTK and GM supervised and performed library preparation for scRNA-Seq. NEG, ZZ, JTM, DNS, EJF, and WJH analyzed scRNA-Seq data. YC analyzed TCGA data. MRL performed the Klickmer assay for mass cytometry. XY and CC acquired mass cytometry data. NEG interpreted data and wrote the initial manuscript. ZZ, JTM, DNS, EJF, and WJH supervised data processing and management. WJH, KMB, DTL, and EMJ conceived the studies. WJH, DTL, EJF, and EMJ funded the studies. All authors contributed to data interpretation and reviewed the manuscript. The order of the 2 first coauthors was based on the relative impact that they had on the development of the project through conception, completion, and writing.

## Supplementary Material

Supplemental data

Unedited blot and gel images

Supporting data values

## Version 1 08/06/2024

In-Press Preview

## Version 2 09/24/2024

Electronic publication
