# Supplementary material for: Phosphodiesterase-5 inhibition collaborates with vaccine-based immunotherapy to reprogram myeloid cells in pancreatic ductal adenocarcinoma
Source: JCI Insight. 2024 Aug 6;9(18):e179292. doi: 10.1172/jci.insight.179292 (PMC11457845; doi:10.1172/jci.insight.179292)
Supplement: Supplemental data [file jciinsight-9-179292-s015.pdf]

Supplemental Tables

Supplemental Table 1. Mass cytometry profiling panel

Mass	Metal	Antigen	Clone	Dilution Factor	Source	Catalogue	Custom
89	Y	PDPN	8.1.8	200	Biolegend®	127402	X
103	Rh	Cell-ID Intercalator-103 Rh	N/A	500	Standard Biotools™	201103A	
106	Cd	CD31	390	200	Biolegend®	102401	X
110	Cd	CD90	G7	400	Biolegend®	105202	X
112	Cd	CD45	30-F11	200	Biolegend®	103102	X
113	Cd	CD45	30-F11	200	Biolegend®	103102	X
114	Cd	CD45	30-F11	200	Biolegend®	103102	X
115	In	CD45	30-F11	200	Biolegend®	103102	X
116	Cd	CD45	30-F11	200	Biolegend®	103102	X
141	Pr	Ly6G	1A8	50	Standard Biotools™	3141008B	
142	Nd	CD11c	N418	100	Standard Biotools™	3142003B	
143	Nd	CD69	H1.2F3	200	Standard Biotools™	3143004B	
144	Nd	CD45R/B220	RA3-6B2	200	Standard Biotools™	3144011B	
145	Nd	CD4	RM4-5	200	Standard Biotools™	3145002B	
146	Nd	F4/80	BM8	100	Standard Biotools™	3146008B	
147	Sm	CD45	30-F11	100	Standard Biotools™	3147003B	
148	Nd	CD11b	M1/70	100	Standard Biotools™	3148003B	
149	Sm	CD19	6D5	100	Standard Biotools™	3149002B	
150	Nd	Ly6C	HK1.4	100	Standard Biotools™	3150010B	
151	Eu	CD25	3C7	100	Standard Biotools™	3151007B	
152	Sm	CD3e	145-2C11	50	Standard Biotools™	3152004B	
153	Eu	CD274/PD-L1	10F.9G2	100	Standard Biotools™	3153031B	
154	Sm	CD152/CTLA-4	UC10-4B9	100	Standard Biotools™	3154008B	
155	Gd	CD134 (OX-40)	OX-86	100	Biolegend®	119430	X
156	Gd	FAP	983810	100	R&D Systems™	MAB97271-100	X
158	Gd	KLRG1	2F1	400	Sigma-Aldrich®	MABF467	X
159	Tb	PD1	29F.1A12	100	Standard Biotools™	3159024B	
160	Gd	Tbet	4B10	100	Standard Biotools™	3160010B	
161	Dy	CD40	HM40-3	50	Standard Biotools™	3161020B	
162	Dy	KI67	B56	200	Standard Biotools™	3162012B	
163	Dy	CD86	GL-1	400	Biolegend®	105002	X
164	Dy	CD62L	MEL14	100	Standard Biotools™	3164003B	
165	Ho	Foxp3	FJK-16s	33	Standard Biotools™	3165024A	
167	Er	EOMES	Dan11mag	200	eBioscience™	14-4875-82	X
168	Er	CD8a	53-6.7	50	Standard Biotools™	3168003B	
169	Tm	CD206	C068C2	100	Standard Biotools™	3169021B	
170	Er	NK1.1	PK136	50	Standard Biotools™	3170002B	
171	Yb	CD44	IM7	300	Standard Biotools™	3171003B	
172	Yb	CD137	17B5	50	Biolegend®	106114	X
173	Yb	Granzyme B	GB11	66	Standard Biotools™	3173006B	
174	Yb	CD223/Lag3	C9B7W	100	Standard Biotools™	3174019B	
175	Lu	MSLN/Mesothelin	B35	100	LS Bio	LS-C179484-100	X
176	Yb	CD278/ICOS	7E.17G9	400	Standard Biotools™	3176014B	
194-198	Pt	Cell-ID Cisplatin	N/A	1000	Standard Biotools™	201064	
209	Bi	I-A/I-E (MHCII)	M5/114.15.2	100	Standard Biotools™	3209006B	

Supplemental Table 2. Estimated cell counts and mean reads per cell by sample for single cell RNA sequencing

Sample	Treatment	Estimated Number of Cells	Mean Reads per Cell
G1mus1	VEH	3,293	240,012
G1mus2	VEH	5,819	134,615
G1mus3	VEH	9,764	85,291
G2mus1	TAD	7,564	97,045
G2mus2	TAD	7,010	111,388
G2mus3rep	TAD	9,759	53,353
G3mus1	CLIST	8,076	98,643
G3mus2	CLIST	22,741	38,090
G3mus3	CLIST	6,863	98,577
G4mus1	MLIST	2,366	294,947
G4mus2	MLIST	6,189	107,831
G4mus3	MLIST	31,210	16,553
G5mus1	MLIST.TAD	4,454	143,383
G5mus2	MLIST.TAD	7,680	66,938
G5mus3	MLIST.TAD	4,442	110,061

Supplemental Figures

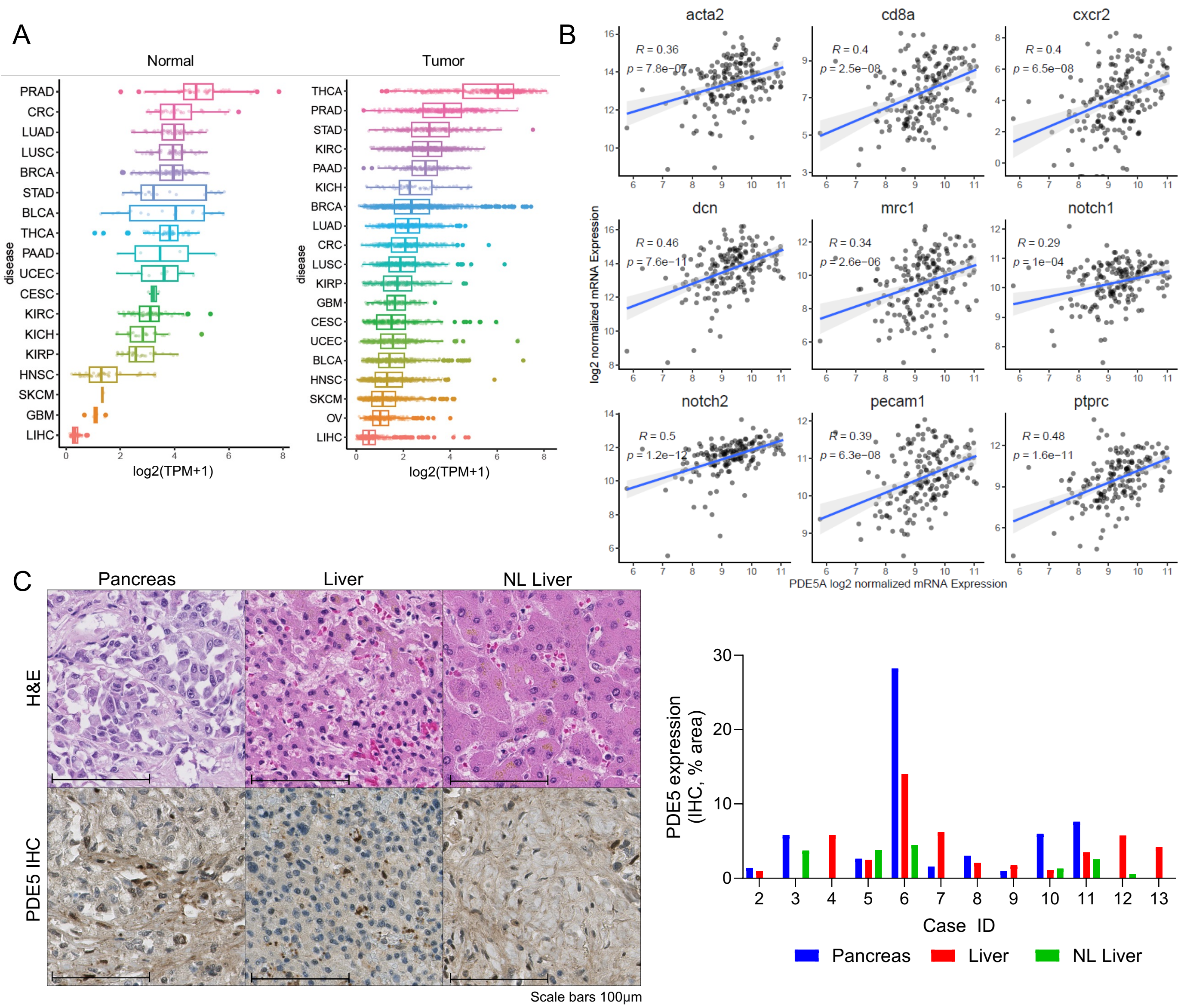

Supplemental Figure 1. Characterization of PDE5 expression in human PDAC. A) Boxplots showing Log2-transformed transcripts per million (TPM) of PDE5A expression in human tissue samples across 19 cancer types and 18 normal tissues. Transcriptome data were derived from The Cancer Genome Atlas (TCGA) Portal. PRAD= prostate adenocarcinoma, CRC= colorectal carcinoma, LUAD= lung adenocarcinoma, LUSC= lung squamous cell carcinoma, BRCA= breast invasive carcinoma, STAD= stomach adenocarcinoma, BLCA= blader urothelial carcinoma, THCA= thyroid carcinoma, PAAD= pancreatic adenocarcinoma, UCEC= uterine corpus endometrial carcinoma, CESC= cervical squamous cell carcinoma and endocervical adenocarcinoma, KIRC= kidney renal clear cell carcinoma, KICH= kidney chromophobe, KIRP= kidney renal papillary cell carcinoma, HNSC= head and neck squamous cell carcinoma, SKCM= skin cutaneous melanoma, GBM= glioblastoma multiforme, LIHC= liver hepatocellular carcinoma, OV= ovarian serous cystadenocarcinoma. **B)** Scatter plots displaying associations between PDE5A log2 normalized mRNA expression and that of various mRNAs. Pearson correlation coefficient was calculated to measure linear correlation. **C)** Representative H&E and PDE5 stained IHC images from 12 human primary pancreas and metastatic liver samples (10 matching), and normal liver tissue samples. Scale bars are 100 μm. Bar plots quantifying the density of PDE5 positively stained tissue area across case IDs.

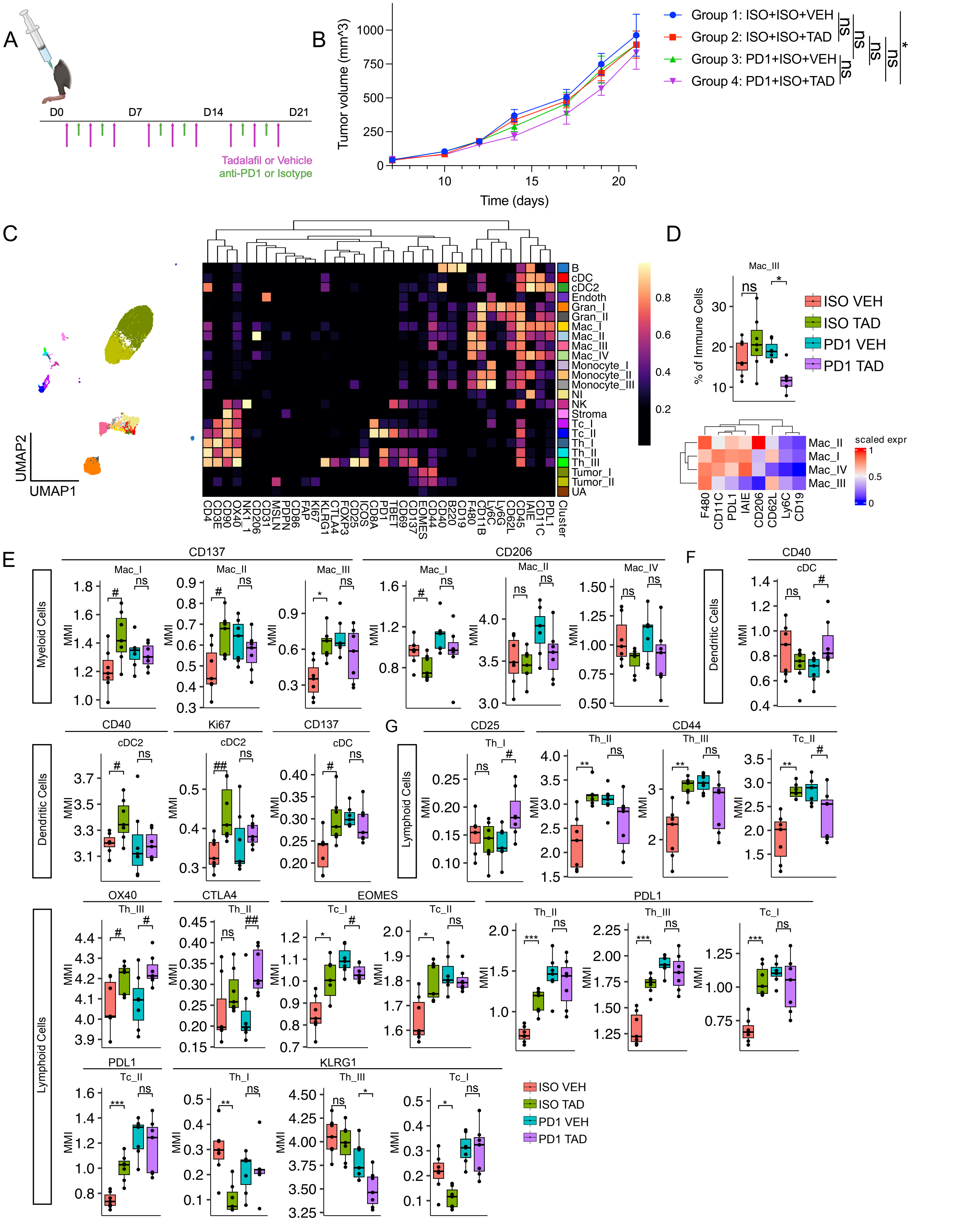

Supplemental Figure 2 Deep profiling of myeloid, dendritic, and lymphoid cells by CyTOF in subcutaneous KPCY 2838c3 model. A) Mice were injected with KPCY 2838c3 cells subcutaneously in the right

hind limb on day 0 then treated intravenously with tadalafil or PBS 3 times per week and intraperitoneally with anti-PD1 or isotype 2 times per week until day 21. **B)** Tumor volumes (mm^3) were measured 3 times per week starting on day 10 and plotted over time for each treatment group. Mean ± SEM (n= 7) **C)** UMAP visualization of annotated cell clusters with 2,000 cells per sample shown and heatmap of final annotated clusters based on relative staining intensities of canonical subtyping markers. B = B cell, cDC = classical dendritic cell, Endoth = endothelial cell, Gran = granulocyte, Mac = macrophage, NI = non-immune, NK = natural killer cell, Tc = cytotoxic T cell, Th = helper T cell, UA = unassigned. **D)** Boxplots showing abundances of IAIE- Macrophage subtype calculated as a percent of all CD45+ cells (n=7). Clustering heatmap showing macrophage subsets based on relative staining intensities of canonical subtyping markers. Mac = macrophage. **E-G)** Boxplots showing mean metal intensity (MMI) of selected functional markers within selected clusters (n=14). For FDR adjusted p values: ns > 0.05, *P ≤ 0.05, **P ≤ 0.001, ***P ≤ 0.001, ****P ≤ 0.0001, for unadjusted p values: #P ≤ 0.05, ##P ≤ 0.001, ###P ≤ 0.001, ####P ≤ 0.0001 by two-tailed Student's T test between groups of interest.

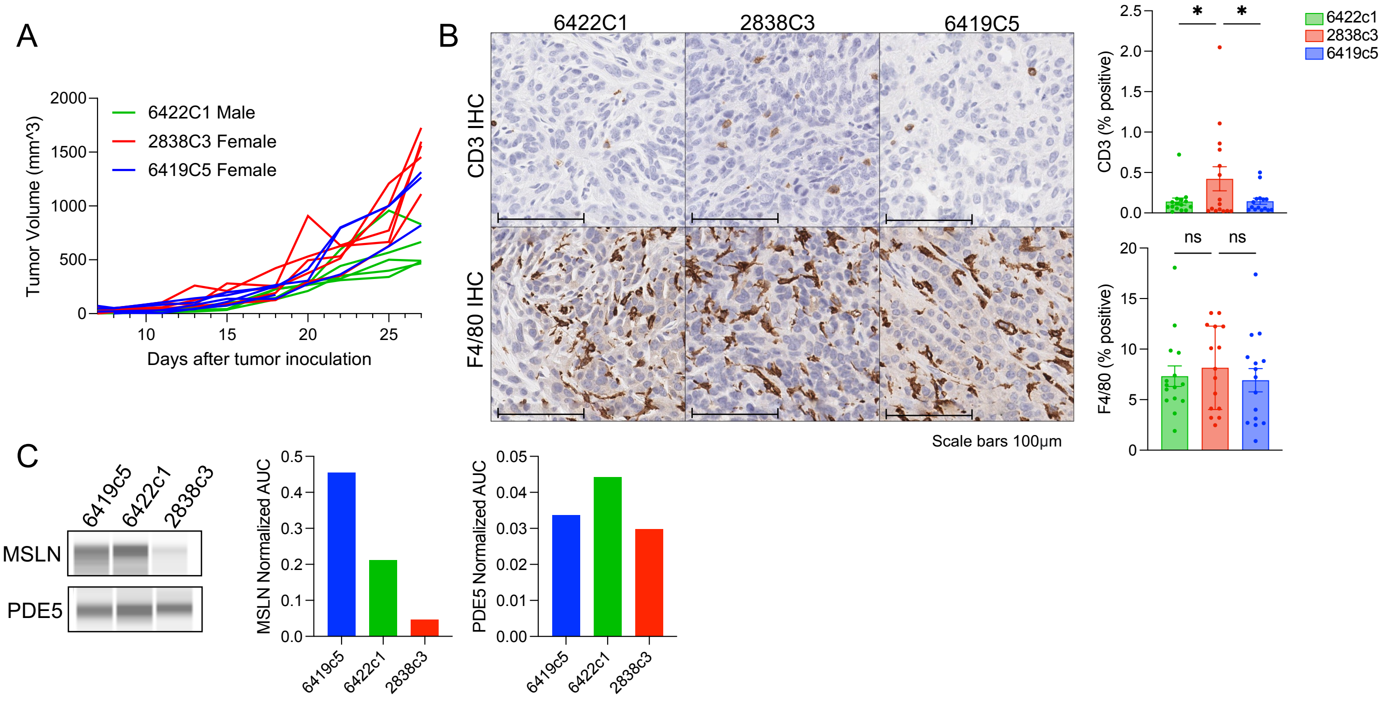

Supplemental Figure 3. Characterization of murine PDAC models. A) Tumor volumes (mm^3) of subcutaneous tumors from 6422c1, 2838c3, and 6419c5 cell lines were measured 3 times per week starting on day 0 and plotted over time for each model for 27 days (n=5). **B)** Representative images and bar graphs of positively stained area for CD3 and F4/80 IHC stains from subcutaneous 6422c1, 2838c3, and 6419c5 tumors. Data represent 3 biological replicates with 5 high powered field images per replicate with mean ± SEM shown. *P ≤ 0.05 by two-tailed Student's T test between groups of interest. **C)** Immunoblot analysis via capillary-based separation of mesothelin (MSLN) and PDE5 expression from 6419c5, 6422c1, and 2838c3 cell lysates with area under the curve (AUC) normalized by total protein plotted for each lane.

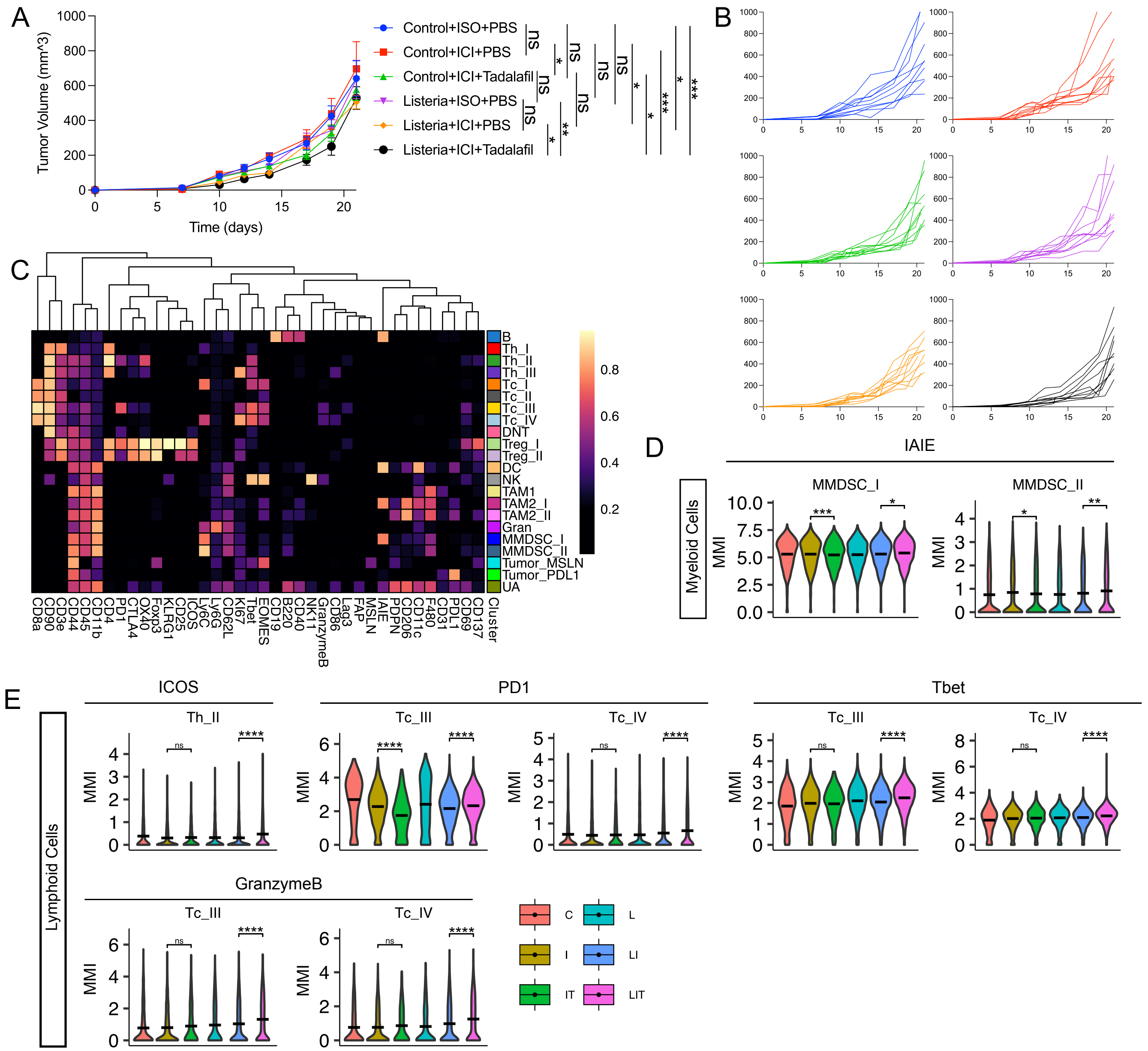

Supplemental Figure 4. Deep profiling of myeloid, dendritic, and lymphoid cells by CyTOF in subcutaneous KPCY 6422c1 model. A) Tumor volumes (mm^3) were measured 3 times per week starting on day 10 and plotted over time for each treatment group. Plot is representative of 2 independent runs. Mean ± SEM (n= 10). ns > 0.05, *P ≤ 0.05, **P ≤ 0.001, ***P ≤ 0.001, ****P ≤ 0.0001 by nonlinear regression. **B)** Tumor volumes plotted individually by treatment group. **C)** Heatmap of final annotated clusters based on relative staining intensities of canonical subtyping markers. B = B cell, Th = helper T cell, Tc = cytotoxic T cell, DNT= double negative T cell, Treg= regulatory T cell, DC = dendritic cell, NK = natural killer cell, TAM = tumor associated macrophage, Gran = granulocyte, MMDSC = monocytic myeloid derived suppressor cell, UA = unassigned. **D-E)** Violin plots showing mean metal intensity (MMI) of selected functional markers within selected clusters. C = control, I = ICIs (anti-PD1 & anti-CTLA-4), IT = ICIs + tadalafil, L = Listeria vaccine, LI = Listeria vaccine + ICIs, LIT = Listeria vaccine + ICIs + tadalafil. For FDR adjusted p values: ns > 0.05, *P ≤ 0.05, **P ≤ 0.001, ***P ≤ 0.001, ****P ≤ 0.0001 by two-tailed Student's T test between groups of interest.

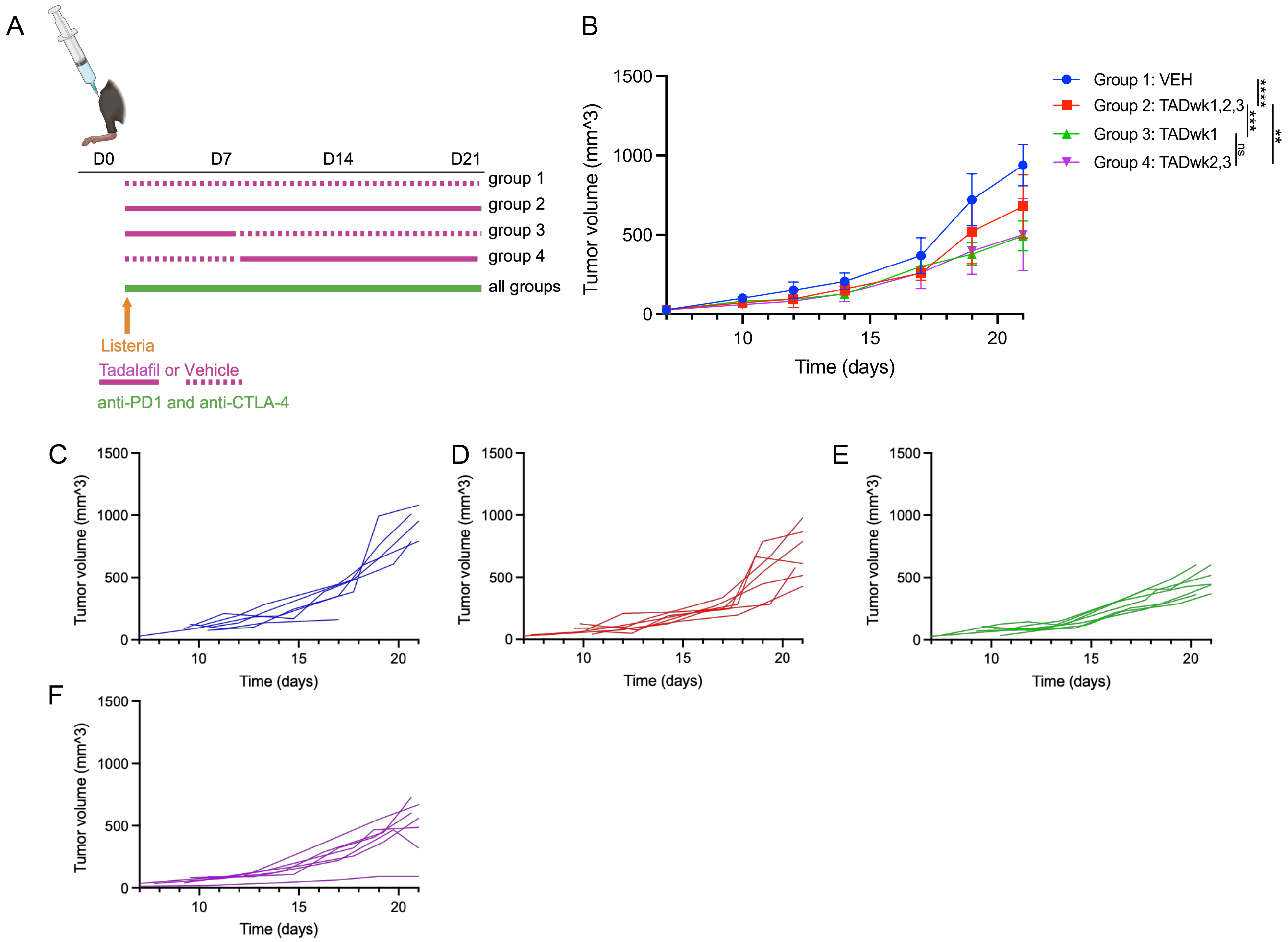

Supplemental Figure 5. The addition of tadalafil to ICIs and mesothelin expressing Listeria vaccine diminishes tumor growth. A) Mice were injected with KPCY 6419c5 cells subcutaneously in the right hind limb on day 0 then vaccinated with mesothelin expressing listeria vaccine on day 3. Mice were treated intravenously with tadalafil or vehicle 3 times per week and intraperitoneally with anti-PD1 & anti-CTLA-4 2 times per week until day 21. All groups received mesothelin expressing listeria vaccine, anti-PD1, and anti-CTLA-4. Group 1 received only vehicle, group 2 received only tadalafil, group 3 received tadalafil for 1 week and vehicle for the following 2 weeks, and group 4 received vehicle for 1 week and tadalafil for the following 2 weeks. **B)** Tumor volumes (mm^3) were measured 3 times per week starting on day 10 and plotted over time for each treatment group. Mean ± SEM (n= 7). ns > 0.05, *P ≤ 0.05, **P ≤ 0.001, ***P ≤ 0.001, ****P ≤ 0.0001 by nonlinear regression. **C-H)** Tumor volumes plotted individually by treatment group.

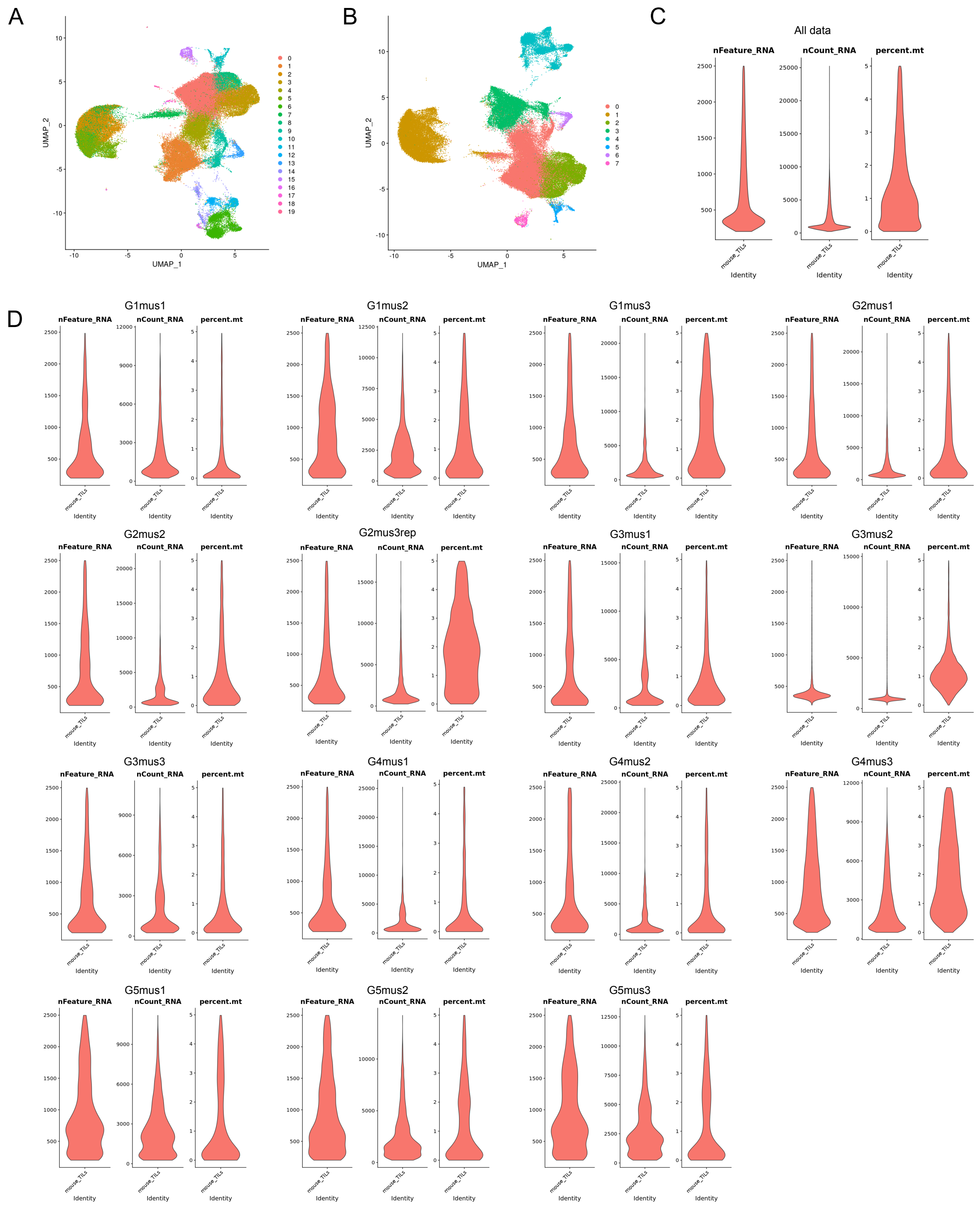

Supplemental Figure 6. scRNAseq quality control. A) Uniform Manifold Approximation and Projection (UMAP) before and **B)** after removing singletons and clusters less than 16 cells. **C)** Violin plots showing unique genes, gene counts, and percent mitochondrial RNA for the total dataset and **D)** per sample.

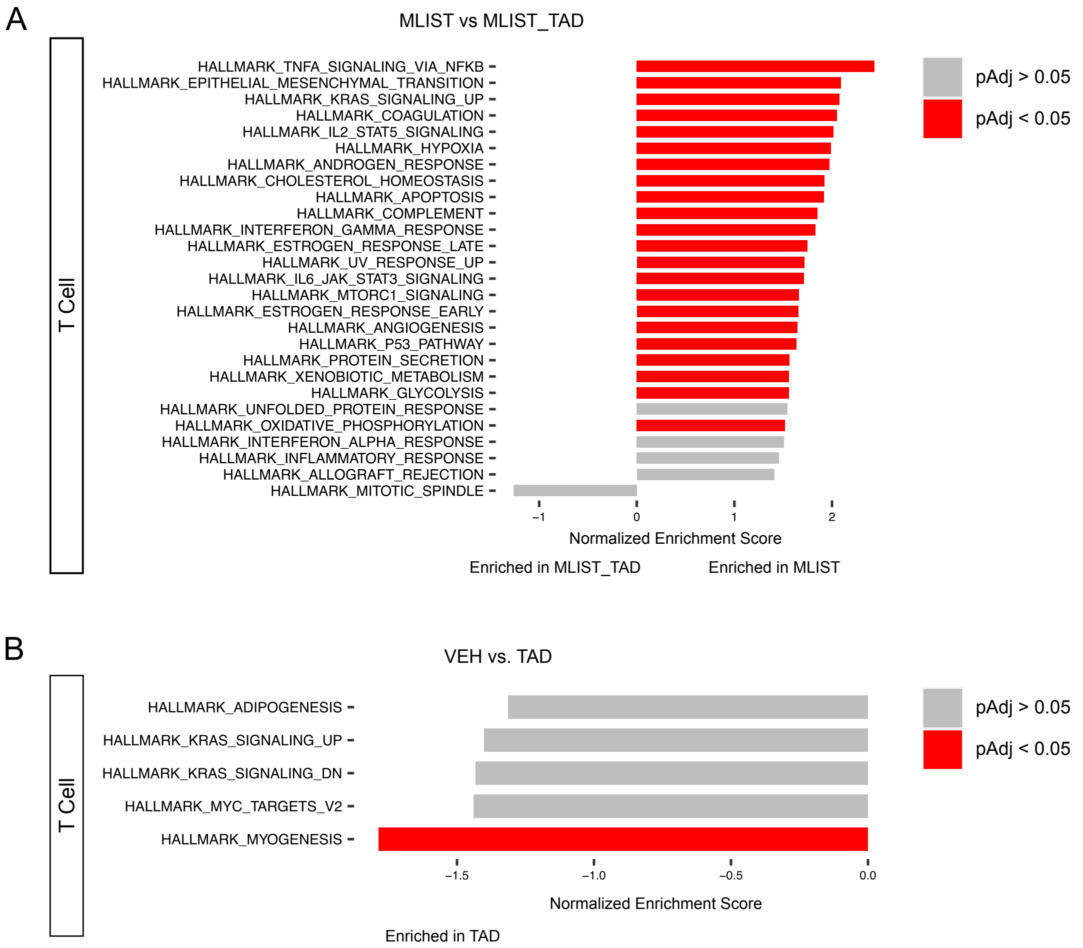

Supplemental Figure 7. Gene set enrichment for T cells. A) Gene set enrichment analysis showing differentially enriched gene sets from the Hallmark database in MLIST vs MLIST_TAD and **B)** VEH vs TAD (n=3). VEH = vehicle, TAD = tadalafil, MLIST = mesothelin listeria, MLIST_TAD = mesothelin listeria + tadalafil.

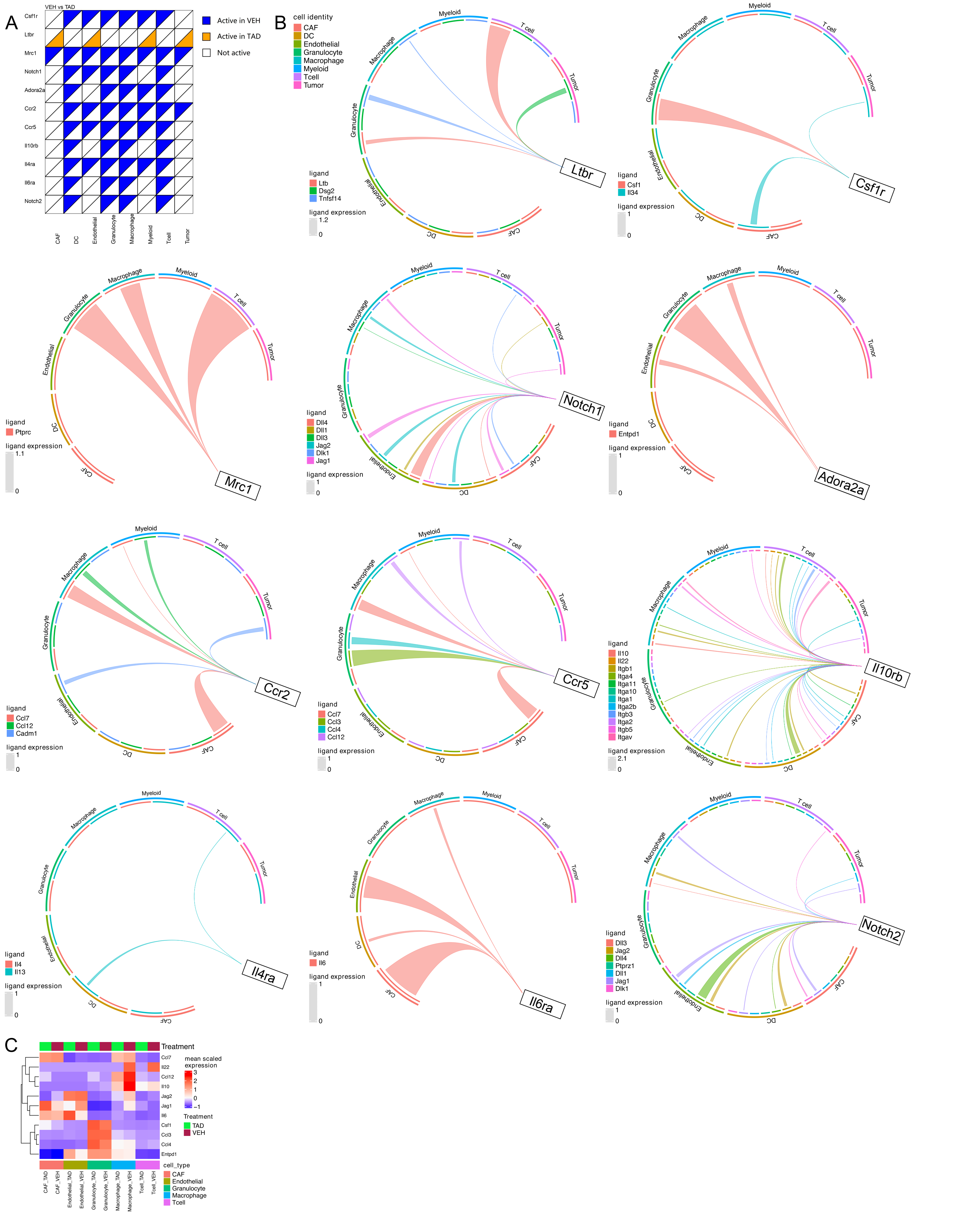

Supplemental Figure 8. Inflammatory signaling axes are active in tadalafil treated groups. A) Binary heatmap showing active receptors in cells by treatment group (n=3). **B)** Circos plots showing ligands of Csf1r, Ltbr, Mrc1, Notch1, Adora2a, Ccl2, Ccl5, Il10rb, Il4ra, Il6ra, Notch2 and their cellular sources. Thickness of lines

correlate to ligand expression levels; thicker lines correspond to higher expression. **C)** Heatmap showing expression levels of selected ligands in cancer associated fibroblasts (CAFs), endothelial cells, granulocytes, macrophages, and T cells.

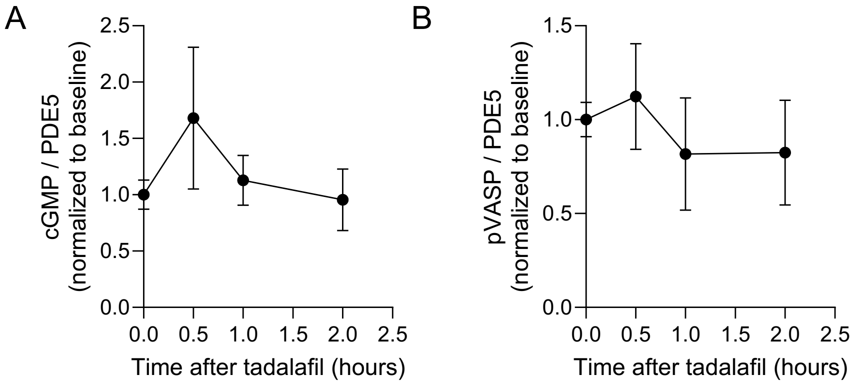

Supplemental Figure 9. Validation of *in vivo* tadalafil inhibition. A) Quantification of cGMP and **B)** phospho-VASP relative to PDE5 in subcutaneous tumors at 0, 0.5, 1, 1.5, and 2 hours post IP injection of tadalafil (n=3). Data represent mean ± SEM.
