# Supplementary material for: Phosphodiesterase-5 inhibition collaborates with vaccine-based immunotherapy to reprogram myeloid cells in pancreatic ductal adenocarcinoma
Source: JCI Insight. 2024 Aug 6;9(18):e179292. doi: 10.1172/jci.insight.179292 (PMC11457845; doi:10.1172/jci.insight.179292)
Supplement: Unedited blot and gel images [file jciinsight-9-179292-s016.pdf]

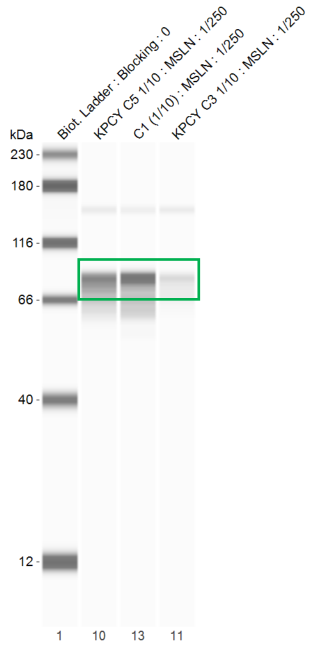

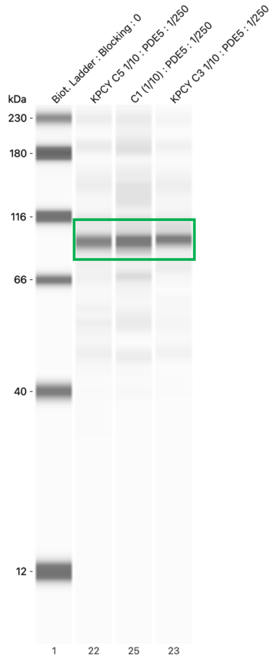

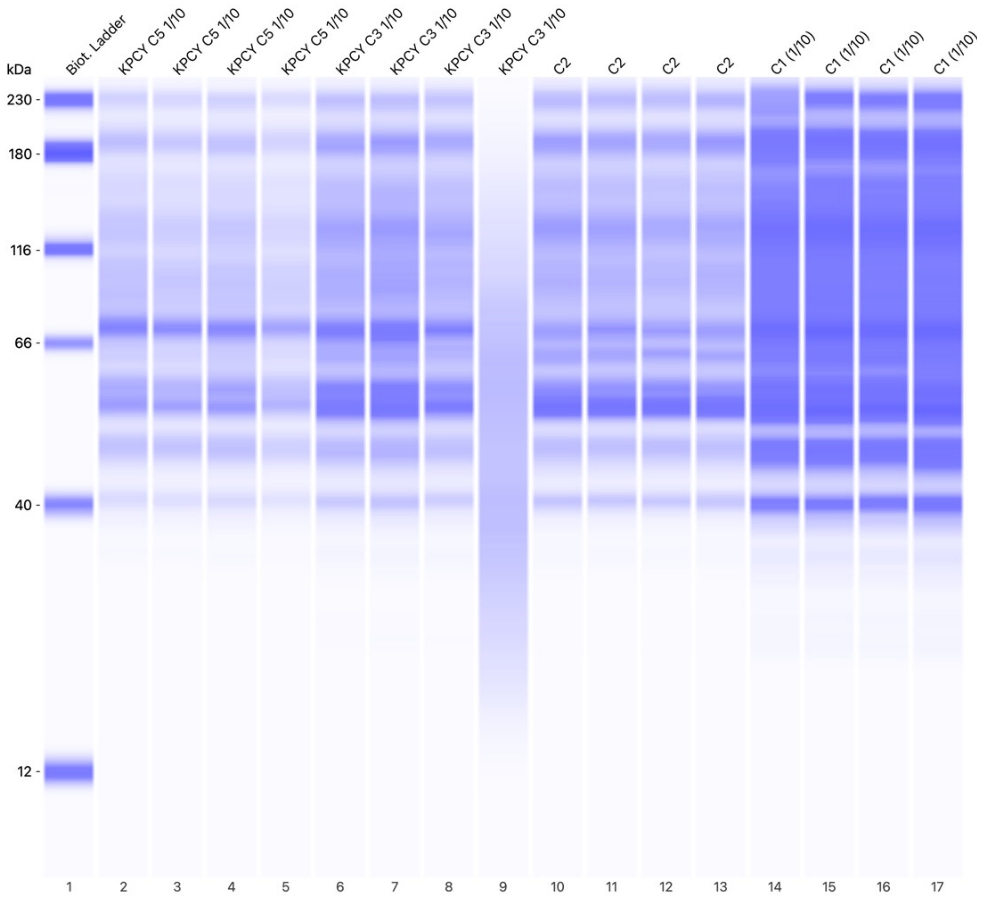

Full unedited image from capillary based separation for Supplemental Figure 3C. Bands used in figure are marked. (Bottom image is from total protein assay)

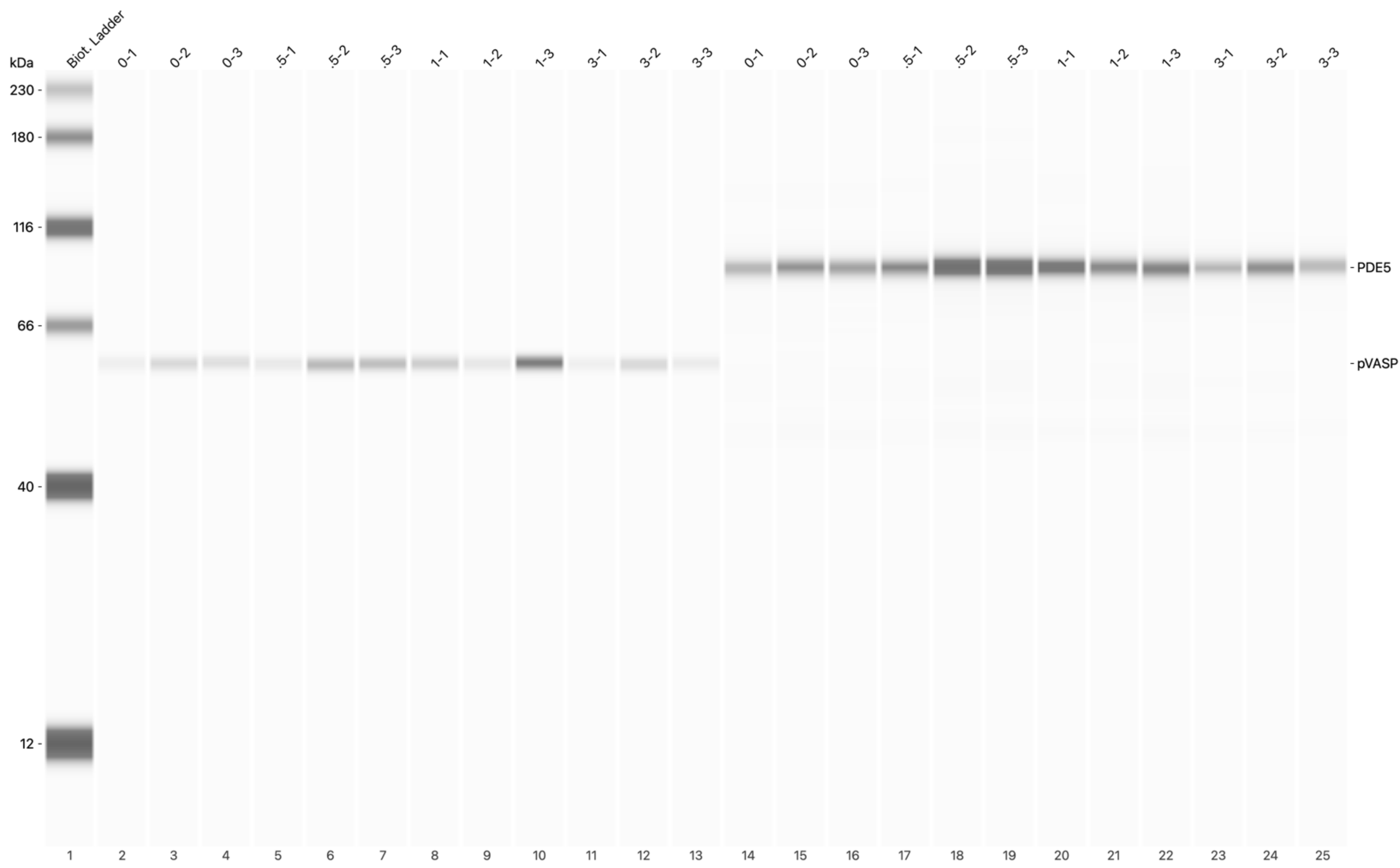

Full unedited image from capillary based separation for Supplemental Figure 9B.
All bands were used in the quantification plotted in the figure.
